# Periodic potentials in hybrid van der Waals heterostructures formed by supramolecular lattices on graphene

**DOI:** 10.1038/ncomms14767

**Published:** 2017-03-21

**Authors:** Marco Gobbi, Sara Bonacchi, Jian X. Lian, Yi Liu, Xiao-Ye Wang, Marc-Antoine Stoeckel, Marco A. Squillaci, Gabriele D'Avino, Akimitsu Narita, Klaus Müllen, Xinliang Feng, Yoann Olivier, David Beljonne, Paolo Samorì, Emanuele Orgiu

**Affiliations:** 1 University of Strasbourg, CNRS, ISIS UMR 7006, 8 Allée Gaspard Monge, F-67000 Strasbourg, France; 2 Laboratory for Chemistry of Novel Materials, Center for Research in Molecular Electronics and Photonics, University of Mons, Place du Parc 20, 7000 Mons, Belgium; 3 Max Planck Institute for Polymer Research, Ackermannweg 10, 55128 Mainz, Germany; 4 Center for Advancing Electronics Dresden (CFAED) and Department of Chemistry and Food Chemistry, Technische Universität Dresden, Mommsenstraße 4, 01062 Dresden, Germany

## Abstract

The rise of 2D materials made it possible to form heterostructures held together by weak interplanar van der Waals interactions. Within such van der Waals heterostructures, the occurrence of 2D periodic potentials significantly modifies the electronic structure of single sheets within the stack, therefore modulating the material properties. However, these periodic potentials are determined by the mechanical alignment of adjacent 2D materials, which is cumbersome and time-consuming. Here we show that programmable 1D periodic potentials extending over areas exceeding 10^4^ nm^2^ and stable at ambient conditions arise when graphene is covered by a self-assembled supramolecular lattice. The amplitude and sign of the potential can be modified without altering its periodicity by employing photoreactive molecules or their reaction products. In this regard, the supramolecular lattice/graphene bilayer represents the hybrid analogue of fully inorganic van der Waals heterostructures, highlighting the rich prospects that molecular design offers to create *ad hoc* materials.

## Abstract (web summary)

Two-dimensional material heterostructures enable unique electronic features by introducing periodic potentials. Here, Gobbi *et al*. use a monolayer supramolecular lattice with a tunable one-dimensional periodic potential to modify the electronic structure of graphene.

Van der Waals (vdW) heterostructures, in which different two-dimensional (2D) materials are superimposed in a stacked configuration, represent a versatile experimental platform to study fundamental properties for device physics and materials science^1^,^2^,^3^. Based on these mechanically assembled stacks, atomically thin novel devices have been demonstrated, such as (tunnelling) transistors^4^, p-n and tunnelling diodes^5^,^6^, photovoltaic elements^7^ and light emitting diodes (LED)^8^.

From a more fundamental point of view, the mutual interaction between vertically-stacked 2D materials generates electronic properties that are different from those of the isolated materials, as experimentally demonstrated for graphene on boron nitride^9^,^10^,^11^,^12^,^13^. In the latter case, a Moiré pattern develops due to the lattice mismatch between the two materials^14^, resulting in a periodic potential (superlattice) with hexagonal geometry capable of modifying profoundly the band structure of graphene. Similarly, other geometries of periodic potentials are predicted to affect the electronic properties of graphene in different ways. For example, a one-dimensional (1D) Kronig–Penney periodic potential with nanoscale periodicity is predicted to create an anisotropic propagation of charge carriers along the different directions of the potential^15^. Hitherto, 1D graphene superlattices that could be pre-programmed with atomic precision have not been demonstrated.

In this context, the use of molecules offers two attractive features. First, organic molecules can prompt doping effects in 2D materials^16^,^17^,^18^,^19^, causing local modifications of their surface potential^20^,^21^,^22^. Second, these molecules form ordered 2D crystalline structures when physisorbed on graphene and other 2D materials^23^,^24^,^25^,^26^,^27^,^28^,^29^,^30^,^31^,^32^,^33^,^34^,^35^. Given the immense opportunities offered by hybrid organic–inorganic vdW heterostructures in modifying the fundamental electronic properties of the pristine materials, the field is still widely unexplored^3^. There are only a few reports connecting doping effects with the specific position of functional groups^26^,^36^,^37^,^38^,^39^, and combining molecules and 2D materials for the technological need of forming p–n junctions^40^,^41^.

In this work, we demonstrate that a tunable periodic potential with 1D geometry on graphene can be realized at ambient conditions in a hybrid vdW heterostructure composed of a 2D supramolecular lattice (SL) self-assembled on graphene, with single domains extending over areas exceeding 250 × 250 nm^2^. The amplitude of the periodic potential is mediated by the specific interaction between graphene and molecular dipoles, while the nanoscale periodicity is determined by the geometry of the self-assembly. In particular, by using a photoreactive organic molecule, we show how a subtle photo-induced change in the chemical structure of the starting molecule leads to a different amplitude of the potential, while leaving the periodicity unaltered.

## Results

### Molecular approach to the formation of periodic potentials

The analogy between hybrid and fully inorganic vdW heterostructures goes beyond their layered nature; in analogy to stacks of inorganic 2D materials, vdW forces drive the interaction between graphene and the self-assembled molecular layer, and similar inter-layer interactions can be envisaged. However, the paradigm leading to the formation of such heterostructure is substantially different. While in inorganic vdW heterostructures the different monolayers are mechanically superimposed with an empirical (optical) alignment^42^, in the molecular case a careful choice of the molecular unit allows the spontaneous creation of self-assembled and ordered layers with predictable geometry and atomic precision. Hence, single molecules can be regarded as molecular building blocks (MBBs) that determine both geometry and functionality of the SL.

**Figure 1:**
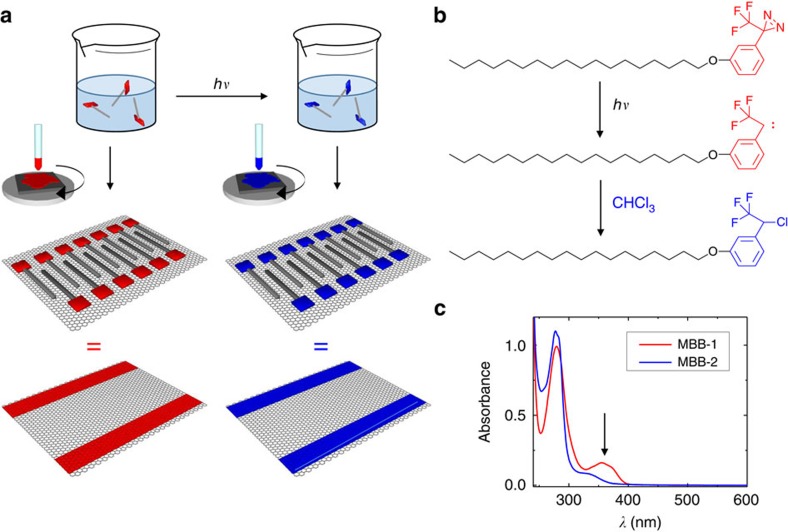
Cartoon of our approach. (**a**) MBB-1 is formed by a linear alkyl tail (sketched as a grey rod) and a photoreactive diazirine head group (red square). The latter undergoes photolysis under exposure to ultraviolet light in chloroform (CHCl_3_) solution forming different reaction products in a mixture (MBB-2) with modified head groups (blue squares). Molecular self-assembly occurs by spin-coating a solution of MBB-1 or MBB-2 on graphene's surface giving rise to supramolecular lattices with identical unit cell for MBB-1 and MBB-2. However, the head groups in MBB-1 and MBB-2 induce different gating effects, resulting in 1D periodic potentials with different amplitude (blue versus red) but with the same 1D periodicity. (**b**) Chemical structures of the molecules. The main reaction product obtained by ultraviolet irradiation in CHCl_3_ is shown in blue, while other products are listed in Supplementary Figs 1–3. (**c**) *In situ* monitoring of the photolysis through ultraviolet–visible spectroscopy (concentration of MBB-1: 2.2 × 10^–3^ M in CHCl_3_; path length: 2 mm (quartz cell); room temperature). In particular, absorption spectra of MBB-1 (red) and MBB-2 (blue) are shown before and after ultraviolet irradiation at *λ=*365 nm for 30 min.

Our approach is schematically illustrated in Fig. 1a. We design an MBB composed of a head, bearing a light-reactive moiety, and a tail, consisting of a long aliphatic chain. This linear tail drives the molecular self-assembly^43^,^44^ and acts as a spacer between adjacent functional heads, forming a lamellar assembly which determines the 1D periodicity of the potential (see Fig. 1a). Electric fields generated by molecular dipoles within the functional heads are responsible for the modulation of the surface potential of graphene, introducing an electric-field effect analogous to that of a constant external gate, and ultimately determining the amplitude and sign of the periodic potentials. Moreover, the functional headgroup of the photoreactive MBB can be modified before deposition on graphene by simple irradiation in different solvents, yielding new MBBs. Specifically, we used 3-trifluoromethyl-3-(3-octadecyloxyphenyl)diazirine (MBB-1), shown in Fig. 1b, as photoreactive MBB. The diazirine headgroup consists of a carbon bound to two nitrogen atoms, and was selected because it can undergo photolysis very efficiently, forming a reactive carbene and nitrogen gas under irradiation with ultraviolet light (Fig. 1b)^45^. If irradiated in chloroform, the *in situ* generated carbene binds to the solvent molecules in its proximity, generating modified MBBs in a mixture which we call MBB-2. As detailed in the Supplementary Figs 1–3, we found that the main reaction product in the mixture has the structure shown in Fig. 1b, in which a Cl atom substitutes the diazirine moiety. This evidence is further corroborated by a joint optical and electrical characterization (see Supplementary Fig. 4) performed on *ad hoc* synthesized molecules (called P-1 and P-2). Absorption spectra allow to follow the change in the molecular head of MBB-1, as shown in Fig. 1c. After 30 min of irradiation at *λ*=365 nm (areal power density 1.7 mW cm^−2^), the characteristic absorption at *λ*=365 nm of MBB-1 disappears, revealing that MBB-1 was efficiently photolyzed.

### Nanoscale characterization of the supramolecular lattices

**Figure 2:**
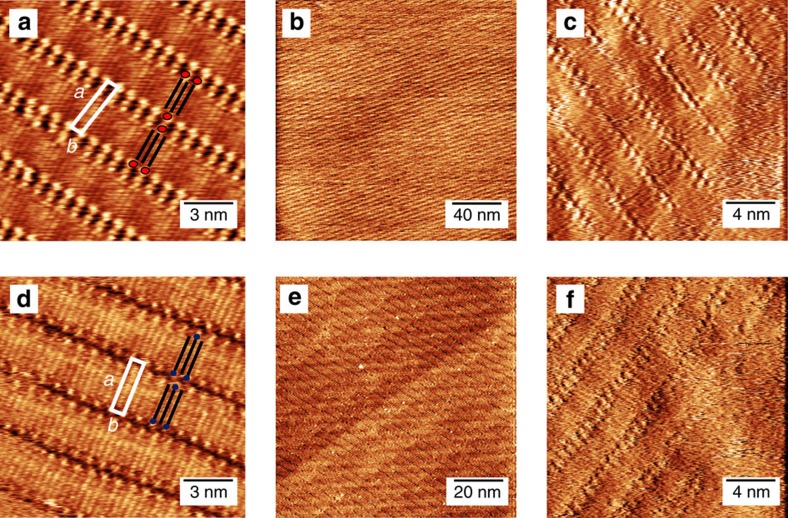
Scanning tunnelling microscopy images of supramolecular lattices. (**a**–**c**) MBB-1 (**d**–**f**) MBB-2. (**a**,**b**,**d**,**e**) Images recorded on highly oriented pyrolytic graphite, the height channel is shown; (**c**,**f**) Images recorded on graphene over SiO_2_, current channel is shown. The images were recorded by using the following tunnelling parameters: (**a**,**b**) tip voltage *V*_t_=400 mV and average tunnelling current *I*_t_=20 pA; (**c**) *V*_t_=−600 mV, *I*_t_=40 pA; (**d**,**e**) *V*_t_=400 mV, *I*_t_=40 pA; and (**f**) *V*_t_=400 mV, *I*_t_=10 pA.

The periodicity and geometry of the SL-induced potential are determined by the nanoscale molecular arrangement which is investigated through scanning tunneling microscope (STM) imaging. We performed STM imaging in ambient conditions on dry SLs, formed by simply spin-coating a solution of either MBB-1 or MBB-2 (as displayed in Fig. 2a–c or 2d–f, respectively). In view of its atomic flatness over hundreds-of-micrometer-sized terraces, the SL can be mapped with increased spatial resolution on highly ordered pyrolitic graphite (HOPG) substrates (Fig. 2a,b,d,e), yet its motif is identical to that obtained when using graphene grown by chemical vapour deposition (CVD) supported on SiO_2_ as substrate (Fig. 2c,f).

The self-assembly is driven by the interplay of molecule–substrate and molecule–molecule interactions; in particular vdW interactions occur between the different alkyl chains, which adsorb flat on graphite/graphene, generating well-defined, crystalline lamellar architectures.

In the case of MBB-1, the functional heads lie close to each other, with the alkyl chains forming an interdigitated structure, exhibiting a unit cell where *a=*(3.8±0.1) nm, *b*=(0.9±0.1)  nm and *α*=(84±2)° therefore leading to an area *A*=(3.4±0.1) nm^2^, with each unit cell containing two molecules (Fig. 2a). Similar SL structures are also observed for MBB-2 (Fig. 2d). Again, the alkyl chains lie flat on the surface by aligning in interdigitated crystalline structures with a similar unit cell where *a*=(3.8±0.2) nm, *b*=(0.9±0.1) nm, *α*=(84±2)° giving rise to an area *A*=(3.5±0.2) nm^2^. The nearly identical unit cells are justified if one considers that on ultraviolet irradiation only a few atoms in the molecular head are replaced. Instead, the long alkyl chains are unaltered in both cases. Hence, MBB-1 and MBB-2 form SLs that possess the same lattice parameters but differ in the head groups, resulting in different electronic interactions with graphene.

For both MBB-1 and MBB-2, crystalline domains with different orientations of the lamellae may form on the graphene surface. This evidence reflects the three-fold symmetry of the substrate and may give rise to misorientation in the direction of the 1D potential. Therefore the size of single-crystalline domains is a key parameter for the relevance of this study on the device physics. To gain detailed insight into this issue, we recorded survey STM images mapping the surface over a scale of a few hundred nanometres. Figure 2b,e show large-area STM images of MBB-1 and MBB-2, respectively. In both cases, an ordered array of lamellae with only one orientation extends over the whole image. Domains with different orientations could be observed in other large-area STM images. After careful analysis of 15 large-area STM images for each MBB, we can conclude that the typical single-domain size is ∼300 nm × 300 nm, as shown in Supplementary Fig. 5 and in Supplementary Table 1. Compared with the length scale relevant for the charge transport in graphene, these single domains are typically two orders of magnitude larger than the typical size of the potential puddles that are responsible for limiting the mobility at the graphene/SiO_2_ interfaces^46^. Moreover, such area is still suitable for nano-fabrication and optical/spectral inspection of the physical properties generated by the presence of the SLs. The assembly of MBB-1 and MBB-2 was investigated also on CVD graphene on SiO_2_ to consider a situation closer to that of the actual devices, as shown in the Fig. 2c,f. In this case, the image resolution is lower, due to the intrinsic roughness of the underlying SiO_2_ substrate. However, even on CVD graphene, it is possible to clearly observe the existence of ordered lamellar structures whose size is in good agreement with that monitored on HOPG. This finding confirms that the MBBs assemble in the same way on both HOPG and graphene/SiO_2_. Large-size STM images show that the crystalline order is maintained over wide areas even on CVD graphene on SiO_2_, as displayed in the Supplementary Fig. 5 for MBB-2. We stress that the STM images in Fig. 2 were measured in air at room temperature and that crystalline domain size and orientation were found to be unchanged even after several hours of continuous acquisition. Moreover, STM images revealed that the unit cell of the molecular assemblies recorded within a few minutes after the SL formation would be identical (within experimental error) to those recorded a few days later. Indeed, the formation of a tight molecular packing along with the strong interaction with graphene promoted by the long alkyl chains would hinder molecular diffusion and thus stabilize the assembly.

### Electrical characterization of devices

**Figure 3:**
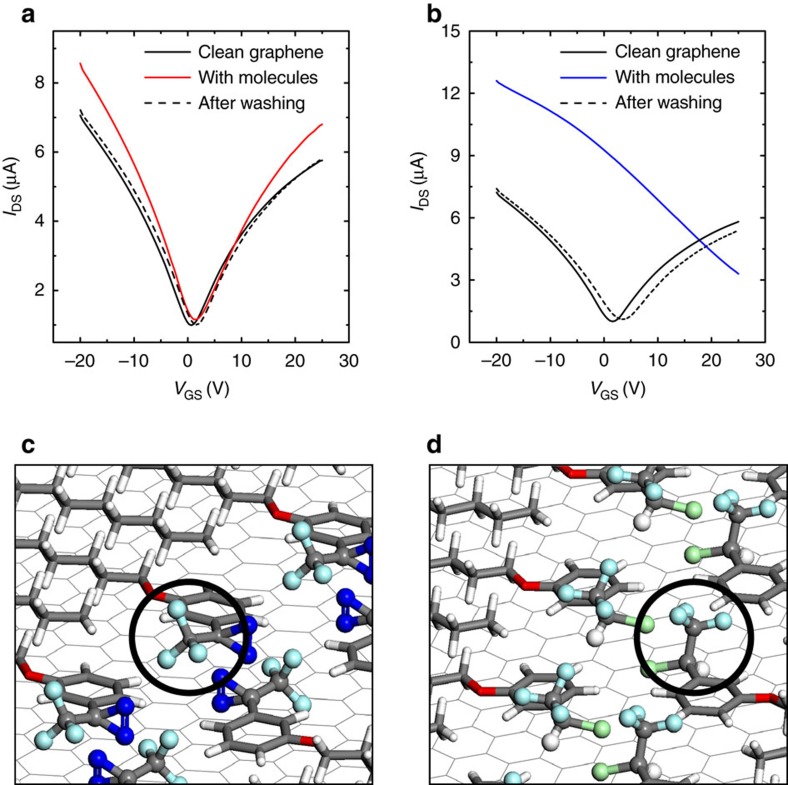
Effect of supramolecular lattices onto the electrical characteristics of graphene. (**a**,**b**) Electrical characteristics of a clean device and of the same device after the formation of MBB-1 (**a**) and MBB-2 (**b**) supramolecular lattices. *I*_DS_ is the drain current, and *V*_GS_ is the gate potential. (**c**,**d**) Zoom of the optimized supramolecular lattice at the functional head groups for (**c**) MBB-1 and (**d**) MBB-2. In **c**,**d**, the position of the CF_3_ groups is highlighted by a black circle. Carbon atoms are shown in grey, hydrogen in white, oxygen in red, nitrogen in dark blue, fluorine in light blue and chlorine in green. As a scale reference, the distance between adjacent carbon atoms in the graphene substrate is 1.4 Å.

To determine whether the molecules are effectively introducing a potential, we study the doping caused by the different SLs on graphene-based field-effect devices (see the ‘Methods' section for details on the device fabrication). The devices were measured before and after the formation of the MBB-1 and MBB-2 SLs, to evaluate their effect on the electrical characteristics of graphene. The measurements were reproduced on three different devices for each MBB, and the results were in qualitative and quantitative agreement (see the ‘Methods' section for details). Representative dependence of the drain current *I*_DS_ on the gate voltage *V*_GS_ is displayed in Fig. 3. In the case of MBB-1 (Fig. 3a), the charge neutrality point was not shifted significantly by the presence of the SL, indicating minor doping effects. Interestingly, the hole mobility increased after the formation of the SL (from 2,200 to 2,650 cm^2^ V^−1^ s^−1^ for the device in Fig. 3a). The increase in mobility was directly related to the presence of the SL, since the initial electrical characteristics of the clean graphene were recovered after the molecules were washed away by rinsing the sample with CHCl_3_, and the mobility decreased to its initial value (see the Fig. 3a).

In the case of MBB-2, after the formation of the SL the charge neutrality point was shifted towards positive voltages (Δ*V*>25 V), as shown in Fig. 3b. This effect corresponds to hole accumulation in the graphene channel, with an induced charge density of Δ*p*>5 × 10^12^ cm^−2^, according to the widely used parallel-plane capacitor model^36^. The electrical characterization provides unambiguous evidence that the effect of the MBB-2 SL–averaged over several randomly oriented domains–is analogous to that of a (fixed) top gate. Moreover, the electrical characterization demonstrates that the doping effect is solely determined by the head group, which is the only part of the MBB modified by the ultraviolet irradiation.

Generally, our devices possess a channel length of a few micrometres that is wider than the typical size of a single domain. As a consequence, our measurements probe not a single but rather a few domains with different random orientations, which impedes measuring the anisotropy in electrical conductance possibly induced by the SL. However, the information extracted by the electrical characterization provides very useful insight on effects, averaged over several randomly oriented domains, associated with the formation of the hybrid vdW heterostructure. While the STM nanoscale characterization provides information about the geometry and periodicity of the potential, the information extracted through device characterization is related to the amplitude of the periodic potential. The demonstration of anisotropy in the conductance of graphene would require a miniaturization of devices that is beyond the scope of this work. To better quantify the effects on the nanoscale electrical properties in presence of the SL, we have performed conductive atomic force microscopy (C-AFM) measurements on a contacted graphene flake, acting as the device active layer, before and after formation of a MBB-1-SL. After formation of the SL, some inhomogeneities in the conductance are introduced with a length scale of a few hundred nanometres, which is the typical size of the single domains imaged by STM. Although the lamellas cannot be visualized by C-AFM, this finding supports the presence of anisotropic conductance within graphene covered by a single-domain SL, as detailed in Supplementary Fig. 6.

### Nanoscale origin of the doping effect

To gain more insight into the origin of the doping effects, the interactions of the molecular assembly with graphene were elucidated through molecular mechanics/molecular dynamics (MM/MD) simulations. For the case of MBB-2, we assumed that all the molecules at the surface possess the structure shown in Fig. 1b, following the findings described in Supplementary Figs 1–4. For both the assembly of MBB-1 and MBB-2, (MM/MD) simulations show that the alkyl chains are interdigitated and the carbon backbones are lying parallel to the graphene surface. The lattice parameters of the calculated unit cell in both cases, that is MBB-1 and MBB-2, are the same, in agreement with the experimental data (see Supplementary Fig. 7). From a closer look at the functional head groups of the calculated assemblies, a major difference appears between the positioning of the molecular head in MBB-1 versus MBB-2 (see Fig. 3c,d). In the former, the diazirine moiety is lying flat on graphene, so that the CF_3_ group is pointing parallel to graphene, whereas the axis of the N=N double bond is normal to the graphene plane (Fig. 3c). Conversely, the CF_3_ unit of MBB-2 is pointing in the *z*-direction normal to the graphene surface (Fig. 3d). Interestingly, such a difference in the orientation of the functional head groups has very limited impact on the overall organization of the molecular adlayer assembly, but it drastically affects the intrinsic dipole moment of the SLs in the *z*-direction. Indeed, in the MBB-1 case the molecules are almost parallel to the graphene surface, so that a small net dipole moment per molecule is expected perpendicular to the surface (Fig. 3c). Instead, in the MBB-2 case, a larger vertical dipole arises from the CF_3_ groups, which are found to be almost perpendicular to the graphene layer (Fig. 3d). Based on this observation, one can qualitatively understand the p-type doping measured in graphene with the MBB-2 adlayer. The electric-field generated by the vertical dipoles acts as a top gate and increases the work function of graphene, effectively inducing p-type doping. At a more quantitative level, density functional theory (DFT) calculations made it possible to estimate the doping induced by the two SLs on the basis of their calculated arrangement, as detailed in Supplementary Methods and Supplementary Figs 8–11. More specifically, molecular doping of graphene is usually sourced by two effects^17^,^47^, namely charge transfer from the molecules, and the presence of molecular dipoles exerting a local gating. DFT calculations allow to disentangle these two contributions for each SL and demonstrate that the charge transfer contribution is almost identical in both cases. Instead, in the case of MBB-1 the calculated vertical molecular dipole is rather low (*μ*_*z*_=−0.17 D mol^−1^), while in the case of MBB-2 it is significantly higher (*μ*_*z*_=−0.82 D mol^−1^). The doping effect in the MBB-2 case, therefore, results from the higher vertical dipole moment of the SLs, as expected from the geometry of the assembly. By accounting for molecular motion at room temperature from MD simulations, we predict a negligible and a significant work function shift for MBB-1 and MBB-2, respectively, as experimentally verified by photoemission spectroscopy in air (see Supplementary Fig. 12). The work function shift is accompanied by an induced p-type doping for MBB-2 (on the order of Δ*p*=5 × 10^12^ cm^−2^) and a minor p-type doping for MBB-1 (Δ*p*<5 × 10^11^ cm^−2^), in excellent agreement with the device experiments. Moreover, our theoretical analysis confirms that the origin of the measured effects can be entirely ascribed to the geometry of the head groups, while the assembly of the alkyl chains, similar for MBB-1 and MBB-2, is not a source of doping.

### Spatially resolved electrostatic potential

Proceeding with the analysis, the spatially resolved electrostatic potential at the graphene/molecule interface was calculated by means of classical microelectrostatic calculations (see Supplementary Methods for details).

**Figure 4:**
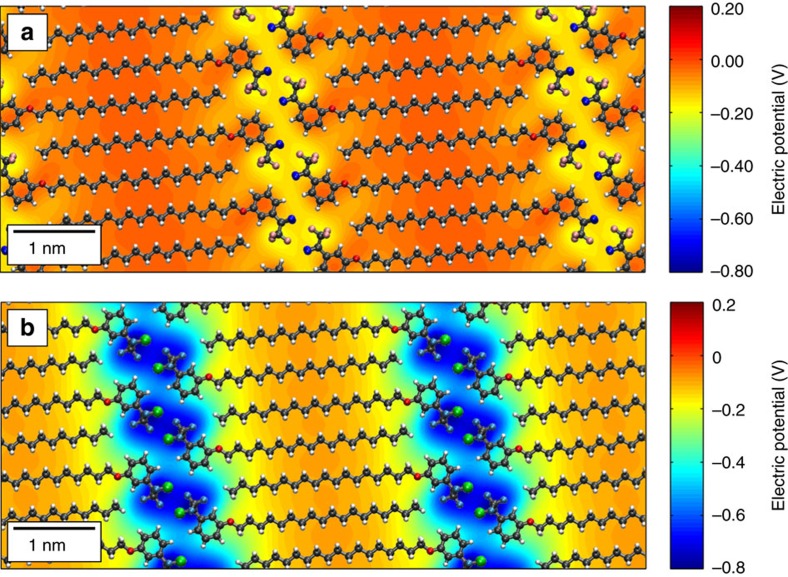
Periodic potentials introduced by the supramolecular lattices. Calculated differential electrical potential induced by a supramolecular lattice of (**a**) MBB-1 and (**b**) MBB-2 on graphene. The supramolecular lattice is superimposed for clarity. The electrical potential is periodically modulated, with negative values in the region below the molecular heads. Carbon atoms are shown in grey, hydrogen in white, nitrogen in red, fluorine in light blue and chlorine in green.

In Fig. 4a,b we show the differential potential *V* (*z*=8 Å)–*V* (*z=*0 Å) for both MBB-1 and MBB-2, where *V* (*z*=8 Å) is the potential calculated on the parallel plane just above the SLs, and *V* (*z*=0 Å) is the potential at the graphene surface. Such differential potential probes the dipolar field associated with the out-of-plane component of the molecular dipoles and has an effect similar to that of a fixed externally applied gate voltage. In both assemblies, such induced gating potential is characterized by a 1D-modulation, with higher negative values localized at the molecular heads separated by inert alkyl chains. The amplitude of the induced gating effect increases by a factor of 4 from MBB-1 to MBB-2, as expected on the basis of the different vertical dipoles. Note that image charge and depolarization effects associated with intermolecular interactions have been estimated using DFT electronic structure calculations implementing periodic boundary conditions. These were found to reduce the electric dipole per molecule by ∼30% for MMB-1 and 20% for MBB-2. Thus, image effects are found to be fairly limited in our case and the graphene layer acts primarily as a template breaking up symmetry in picking selected dipolar conformations for the MBBs at the surface, which then translates into the observed electrostatic potential shown in Fig. 4.

This different amplitude induced by the MBB-1 and MBB-2 SL demonstrates the ability of introducing significant variations in the SL-induced periodic grating with a subtle light-induced change in the molecular structure. In both cases, the in-plane distribution of the potential can be considered as a nanoscopic experimental realization of a Kronig–Penney potential. We highlight that not only its geometry, but also its amplitude is within the same order of magnitude of that considered in the initial prediction of anisotropic behaviour of charge carriers in graphene superlattices^15^.

### Modification of the periodic potential

**Figure 5:**
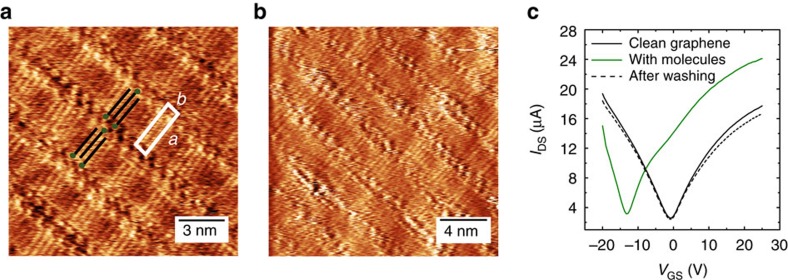
Modification of the periodic potential. Scanning tunnelling microscopy images of the nanoscale assembly of MBB-3 on different substrates: (**a**) on highly oriented pyrolytic graphite (height channel) and (**b**) on graphene over SiO_2_ (current channel). The images were recorded by using the following tunnelling parameters: (**a**) *V*_t_=−400 mV, *I*_t_=20 pA; (**b**) *V*_t_=600, *I*_t_=60 pA. The effect of the SL formed by MBB-3 on the electrical characteristics of a graphene device is shown in **c**. *I*_DS_ is the drain current, and *V*_GS_ is the gate potential.

Finally, we show that periodic potentials with the same geometry but different intensity can be achieved by preparing SLs of MBB-1 after ultraviolet irradiation in different solvents. To prove further this concept, we present here a detailed nanoscale and electrical characterization of MBB-1 after ultraviolet irradiation in diethylamine, which results in a mixture hereafter referred to as MBB-3. The assembly of MBB-3 is shown in Fig. 5a,b on HOPG and CVD graphene, respectively. Similarly to MBB-1 and MBB-2, MBB-3 self-assembles forming ordered lamellae in which the functional heads lie close to each other, with the alkyl chains forming an interdigitated structure. The unit cell parameters were *a=*(3.8±0.2) nm, *b*=(0.9±0.1) nm and *α*=(84±2)° which lead to an area *A*=(3.5±0.2) nm^2^, analogous to that of MBB-1 and MBB-2. Again, the atoms incorporated through photolysis do not perturb the assembly at the nanoscale, which is determined and imposed by the alkyl chains. Instead, the intensity of the induced potential is determined by the interaction with the head groups. The overall effect can be measured in a three-terminal device, by covering a graphene device with a MBB-3 SL. In this case (Fig. 5c), the charge neutrality point shifts towards negative values, corresponding to electron accumulation (n-doping) in the graphene channel. A shift in the charge neutrality point Δ*V*=−11 V corresponds to an induced electron density Δ*n*=2.6 × 10^12^ cm^−2^. Even in this case, the electrical properties of the clean graphene are recovered when the molecules are washed away from the graphene surface with a simple rinsing step in chloroform.

Interestingly, the effect on the *I*_DS_−*V*_GS_ traces of graphene is opposite to that of MBB-2. Since the induced periodic potential is primarily determined by the orientation of local molecular dipoles, one is thus led to conclude that the sign of such a potential for MBB-3 is opposite to that of MBB-2.

## Discussion

The concept that interplanar interactions are capable of modifying fundamental electronic properties of pristine materials lies at the heart of the research field of vdW heterostructures. In this context, the possibility of introducing tunable 1D potentials through organic SLs is particularly appealing. For instance, one could combine graphene covered by a SL with other inorganic 2D materials to fabricate more complex multi-layered hybrid vdW heterostructures. In such systems, an ordered lamellar assembly modifying the momentum dispersion of graphene would induce anisotropy not only on the in-plane (graphene) but also on the inter-layer charge transport, for which momentum conservation plays an important role^48^. Moreover, a fully anisotropic heterostructure might be obtained by combining our SL-covered graphene with other 2D materials intrinsically possessing 1D anisotropy (such as ReS_2_ (ref. 49) and black phosphorus^50^) to demonstrate novel device architectures actively exploiting charge transport along a preferential direction. Finally, we point out that bottom–up supramolecular approaches for the realization of periodic potentials is not limited to graphene, since analogous molecules form ordered 1D assemblies also on the surface of other 2D materials^51^, widening the horizons of the present study. For instance, a SL similar to those described in this study assembled on semiconducting transition metal dichalcogenides (such as WSe_2_ and MoS_2_) might introduce a 1D periodic potential capable of locally varying the position of the Fermi level, resulting in alternating hole- or electron- rich regions. In this way, one would obtain a series of consecutive nanoscale p–n junctions with distinctive charge transport properties and optical response.

In conclusion, our study shows that organic SLs represent an ideal system for generating periodic potentials whose periodicity, amplitude and sign can be pre-programmed by careful molecular design. In particular, here we have shown that a periodic potential with 1D geometry can be generated at the graphene surface and manipulated by making use of molecular photo-reactivity. In perspective, a 1D potential will offer the opportunity to introduce anisotropy in otherwise isotropic materials, hence paving the way for design and implementation of novel functional vdW heterostructures, for example, for 1D charge transport. The control over periodic potentials can hardly be achieved in vdW heterostructures based solely on inorganic 2D materials, while their combination with SLs allows expanding substantially the 2D material library. Chemistry can offer an almost unlimited choice of SL that can form hybrid vdW heterostructures with controllable structural and electronic properties, exerting periodic potentials with tunable amplitude and periodicity. In perspective, a great deal of novel electrical, magnetic, piezoelectric and optical functionalities arising from such hybrid vdW heterostructures are expected by taking full advantage of the infinite degrees of freedom offered by the design of the MBBs.

## Methods

### Sample preparation

Full details regarding the characterization and synthesis of the MBBs are given in Supplementary Figs 13–19 and in Supplementary Note 1.

### Ultraviolet–visible spectroscopy

Absorption spectra were recorded at room temperature (∼25 °C) with a JASCO V-670 spectrophotometer and all solutions were examined in quartz cells with 2-mm pathlength (HELLMA) with a concentration of 2.2 × 10^–3^ M. Photochemical reaction of MBB-1 was performed in air-equilibrated CHCl_3_ (Uvasol Merck-Millipore) or in degassed diethyalmine (Sigma-Aldrich) by using an Ultraviolet lamp (UV-6 L/M Herolab) with *λ*_irr_=365 (±5) nm and a power density=1.7 mW cm^−2^.

### Device fabrication and characterization

Back gated devices were fabricated on Si/SiO_2_ (90 nm) substrates with a Microtech laser writer, exposing with a *λ*=405 nm laser a standard photoresist (AZ1505, Microchemicals). Gold (without adhesion layer) was thermally evaporated onto the patterned photoresist and lift-off was carried out in warm acetone (40 °C). After fabrication, the devices were immersed in warm NMP (40 °C) overnight, rinsed with chloroform, acetone and isopropanol. The devices were kept in a nitrogen-filled glove box in which they could be measured in a probe station connected to a Keithley 2636. By employing this procedure, the graphene device showed almost-ideal, symmetric and stable current–voltage *I*_DS_−*V*_GS_ characteristics, with the charge neutrality point close to a gate voltage *V*_GS_=0 V, and hole mobility reproducibly above 1,800 cm^2^ V^−1^ s^−1^. The carrier mobility was determined by the conventional parallel-plane capacitor model:


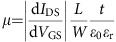


where *L* and *W* are the graphene channel length and width, *t* is the thickness of gate oxide, *ɛ*_r_ is the relative dielectric permittivity of SiO_2_. The mobility was extracted at the carrier concentration value of |*n*|=1 × 10^12^ cm^−2^, by measuring d*I*_DS_/d*V*_GS_ at 4.5 V away from the charge neutrality point. In all the electrical measurements, the potential applied between the graphene electrodes was *V*=10 mV. The reproducibility of the effect of each SL on the electrical characteristics of devices was tested by repeating the measurement on three different devices for each MBB. For every test, a fresh solution of MBB was employed. The doping effect is reproducible: MBB-1 was found to induce small p-type doping (Δ*V*<5 V in the three tested devices); MBB-2 significantly higher p-type doping (Δ*V*>25 V in the three tested devices); and MBB-3 n-type doping (Δ*V* in the range between −7 and −12 V in the three tested devices). Moreover, the experiments were repeated several times on the same device, by washing the MBBs away and re-forming the SL. In the cases of MBB-1 and MBB-3, the increase in mobility after the formation of the SL was also found in the three tested devices.

### Supramolecular lattice formation

To form the SLs, molecules were spin-coated onto either HOPG or CVD graphene on SiO_2_ and onto devices from chloroform solutions (1 mg ml^−1^). For the sake of consistency, the solutions were spin-coated onto the graphene devices by employing the same parameters used for the STM imaging. In the case of irradiation in diethylamine, the molecules were dried after irradiation and re-solubilized in chloroform before spin-coating. In the case of devices, the solution were spin-coated *in situ* in the nitrogen-filled glovebox. The SLs could be washed away by rinsing the substrates or the devices with CHCl_3_.

### Scanning tunneling microscopy

STM measurements were carried out by using a Veeco scanning tunneling microscope (multimode Nanoscope III, Veeco) operating with an A piezoelectric scanner which allowed the mapping of a maximum area of 1 μm × 1 μm. As substrates, we used highly oriented pyrolytic graphite and commercial CVD graphene supported on Si/SiO_2_ (300 nm) purchased from Graphenea. The graphene sample has been used as received, without any additional cleaning step, and has been stored in air for 4 months before the STM experiments. The substrates were glued onto a magnetic disk and an electric contact was made with (conductive) silver paint (Aldrich Chemicals).

The STM tips were mechanically cut from a Pt/Ir wire (90/10, diameter 0.25 mm). The images were obtained in air at room temperature. The raw STM data were processed through the application of background flattening, and in the case of the HOPG substrates in Figs 2a,d and 5a the drift of the piezo was corrected using the underlying graphite lattice as a reference. The lattice of the underlying substrate was visualized by lowering the bias voltage *V*_t_ to 10 mV and setting the average tunneling current *I*_t_=60 pA. Tip height and current were measured for all STM images.

### MD/MM and DFT calculations

Full details regarding the MD/MM and DFT calculations are given in Supplementary Methods and in Supplementary Table 2.

### Data availability

The data that support the findings of this study are available from the corresponding authors on request.

## Additional information

**How to cite this article:** Gobbi, M. *et al*. Periodic potentials in hybrid van der Waals heterostructures formed by supramolecular lattices on graphene. *Nat. Commun.* **8,** 14767 doi: 10.1038/ncomms14767 (2017).

**Publisher's note**: Springer Nature remains neutral with regard to jurisdictional claims in published maps and institutional affiliations.

## Supplementary Material

Supplementary Information Supplementary Figures, Supplementary Tables, Supplementary Methods, Supplementary Note and Supplementary References.

## References

[b1] Geim A. K. & Grigorieva I. V. Van der Waals heterostructures. Nature 499, 419–425 (2013). 23887427 10.1038/nature12385

[b2] Novoselov K. S., Mishchenko A., Carvalho A. & Castro Neto A. H. 2D materials and van der Waals heterostructures. Science 353, aac9439 (2016). 27471306 10.1126/science.aac9439

[b3] Jariwala D., Marks T. J. & Hersam M. C. Mixed-dimensional van der Waals heterostructures. Nat. Mater. 16, 170–181 (2017). 27479211 10.1038/nmat4703

[b4] Britnell L. . Field-effect tunneling transistor based on vertical graphene heterostructures. Science 335, 947–950 (2012). 22300848 10.1126/science.1218461

[b5] Lee C.-H. . Atomically thin p–n junctions with van der Waals heterointerfaces. Nat. Nanotechnol. 9, 676–681 (2014). 25108809 10.1038/nnano.2014.150

[b6] Britnell L. . Resonant tunnelling and negative differential conductance in graphene transistors. Nat. Commun. 4, 1794 (2013). 23653206 10.1038/ncomms2817 PMC3644101

[b7] Britnell L. . Strong light-matter interactions in heterostructures of atomically thin films. Science 340, 1311–1314 (2013). 23641062 10.1126/science.1235547

[b8] Withers F. . Light-emitting diodes by band-structure engineering in van der Waals heterostructures. Nat. Mater. 14, 301–306 (2015). 25643033 10.1038/nmat4205

[b9] Ponomarenko L. A. . Cloning of Dirac fermions in graphene superlattices. Nature 497, 594–597 (2013). 23676678 10.1038/nature12187

[b10] Dean C. R. . Hofstadter's butterfly and the fractal quantum Hall effect in moiré superlattices. Nature 497, 598–602 (2013). 23676673 10.1038/nature12186

[b11] Hunt B. . Massive Dirac fermions and Hofstadter butterfly in a van der Waals heterostructure. Science 340, 1427–1430 (2013). 23686343 10.1126/science.1237240

[b12] Gorbachev R. V. . Detecting topological currents in graphene superlattices. Science 346, 448–451 (2014). 25342798 10.1126/science.1254966

[b13] Wang L. . Evidence for a fractional fractal quantum Hall effect in graphene superlattices. Science 350, 1231–1234 (2015). 26785484 10.1126/science.aad2102

[b14] Yankowitz M. . Emergence of superlattice Dirac points in graphene on hexagonal boron nitride. Nat. Phys. 8, 382–386 (2012).

[b15] Park C.-H., Yang L., Son Y.-W., Cohen M. L. & Louie S. G. Anisotropic behaviours of massless Dirac fermions in graphene under periodic potentials. Nat. Phys. 4, 213–217 (2008). 10.1103/PhysRevLett.101.126804 18851401

[b16] Schedin F. . Detection of individual gas molecules adsorbed on graphene. Nat. Mater. 6, 652–655 (2007). 17660825 10.1038/nmat1967

[b17] Wehling T. O. . Molecular doping of graphene. Nano Lett. 8, 173–177 (2008). 18085811 10.1021/nl072364w

[b18] Liu H., Liu Y. & Zhu D. Chemical doping of graphene. J. Mater. Chem. 21, 3335–3345 (2011).

[b19] Samuels A. J. & Carey J. D. Molecular doping and band-gap opening of bilayer graphene. ACS Nano 7, 2790–2799 (2013). 23414110 10.1021/nn400340q

[b20] Cervenka J. . Graphene field effect transistor as a probe of electronic structure and charge transfer at organic molecule-graphene interfaces. Nanoscale 7, 1471–1478 (2014). 10.1039/c4nr05390g 25502349

[b21] Hapala P. . Mapping the electrostatic force field of single molecules from high-resolution scanning probe images. Nat. Commun. 7, 11560 (2016). 27230940 10.1038/ncomms11560 PMC4894979

[b22] Wickenburg S. . Tuning charge and correlation effects for a single molecule on a graphene device. Nat. Commun. 7, 13553 (2016). 27886170 10.1038/ncomms13553 PMC5133630

[b23] Prado M. C. . Two-dimensional molecular crystals of phosphonic acids on graphene. ACS Nano 5, 394–398 (2011). 21186832 10.1021/nn102211n

[b24] Wang Q. H. & Hersam M. C. Room-temperature molecular-resolution characterization of self-assembled organic monolayers on epitaxial graphene. Nat. Chem. 1, 206–211 (2009). 21378849 10.1038/nchem.212

[b25] Deshpande A. . Self-assembly and photopolymerization of sub-2 nm one-dimensional organic nanostructures on graphene. J. Am. Chem. Soc. 134, 16759–16764 (2012). 22928587 10.1021/ja307061e

[b26] Zhang T. . Self-assembled 1-octadecanethiol monolayers on graphene for mercury detection. Nano Lett. 10, 4738–4741 (2010). 20931998 10.1021/nl1032556

[b27] Järvinen P. . Molecular self-assembly on graphene on SiO_2_ and h-BN substrates. Nano Lett. 13, 3199–3204 (2013). 23786613 10.1021/nl401265f

[b28] Riss A. . Imaging and tuning molecular levels at the surface of a gated graphene device. ACS Nano 8, 5395–5401 (2014). 24746016 10.1021/nn501459v PMC4070845

[b29] Tsai H.-Z. . Molecular self-assembly in a poorly screened environment: F_4_TCNQ on graphene/BN. ACS Nano 9, 12168–12173 (2015). 26482218 10.1021/acsnano.5b05322 PMC4690193

[b30] Mali K. S., Greenwood J., Adisoejoso J., Phillipson R. & De Feyter S. Nanostructuring graphene for controlled and reproducible functionalization. Nanoscale 7, 1566–1585 (2015). 25553734 10.1039/c4nr06470d

[b31] Alaboson J. M. P. . Templating sub-10 nm atomic layer deposited oxide nanostructures on graphene via one-dimensional organic self-assembled monolayers. Nano Lett. 13, 5763–5770 (2013). 23464881 10.1021/nl4000932

[b32] Macleod J. M. & Rosei F. Molecular self-assembly on graphene. Small 10, 1038–1049 (2014). 24155272 10.1002/smll.201301982

[b33] Zeng M. . Self-assembly of graphene single crystals with uniform size and orientation: the first 2D super-ordered structure. J. Am. Chem. Soc. 138, 7812–7815 (2016). 27313075 10.1021/jacs.6b03208

[b34] Huang H., Chen S., Gao X., Chen W. & Wee A. T. S. Structural and electronic properties of PTCDA thin films on epitaxial graphene. ACS Nano 3, 3431–3436 (2009). 19852489 10.1021/nn9008615

[b35] Zhou H. T. . Direct imaging of intrinsic molecular orbitals using two-dimensional, epitaxially-grown, nanostructured graphene for study of single molecule and interactions. Appl. Phys. Lett. 99, 2009–2012 (2011).

[b36] Li B. . Toward tunable doping in graphene FETs by molecular self-assembled monolayers. Nanoscale 5, 9640–9644 (2013). 23827941 10.1039/c3nr01255g

[b37] Zhang X. . Supramolecular chemistry on graphene field-effect transistors. Small 10, 1735–1740 (2014). 24515931 10.1002/smll.201303098

[b38] Yu Y. . Epitaxially self-assembled alkane layers for graphene electronics. Adv. Mater. 29, 1603925 (2017). 10.1002/adma.201603925 27905154

[b39] Phillipson R. . Tunable doping of graphene by using physisorbed self-assembled networks. Nanoscale 8, 20017–20026 (2016). 27883146 10.1039/c6nr07912a

[b40] Vélez S. . Gate-tunable diode and photovoltaic effect in an organic–2D layered material p–n junction. Nanoscale 7, 15442–15449 (2015). 26335856 10.1039/c5nr04083c

[b41] Jariwala D. . Hybrid, Gate-tunable, van der Waals p−n heterojunctions from pentacene and MoS_2_. Nano Lett. 16, 497–503 (2016). 26651229 10.1021/acs.nanolett.5b04141

[b42] Castellanos-Gomez A. . Deterministic transfer of two-dimensional materials by all-dry viscoelastic stamping. 2D Mater. 1, 011002 (2014).

[b43] De Feyter S. & De Schryver F. C. Self-assembly at the liquid/solid interface: STM reveals. J. Phys. Chem. B 109, 4290–4302 (2005). 16851494 10.1021/jp045298k

[b44] De Feyter S. & De Schryver F. C. Two-dimensional supramolecular self-assembly probed by scanning tunneling microscopy. Chem. Soc. Rev. 32, 139–150 (2003). 12792937 10.1039/b206566p

[b45] Lawrence E. J. . 3-Aryl-3-(trifluoromethyl)diazirines as versatile photoactivated ‘linker' molecules for the improved covalent modification of graphitic and carbon nanotube surfaces. Chem. Mater. 23, 3740–3751 (2011).

[b46] Zhang Y., Brar V. W., Girit C., Zettl A. & Crommie M. F. Origin of spatial charge inhomogeneity in graphene. Nat. Phys. 5, 722–726 (2009).

[b47] Wehling T. O., Lichtenstein A. I. & Katsnelson M. I. First-principles studies of water adsorption on graphene: the role of the substrate. Appl. Phys. Lett. 93, 202110 (2008).

[b48] Massicotte M. . Photo-thermionic effect in vertical graphene heterostructures. Nat. Commun. 7, 12174 (2016). 27412308 10.1038/ncomms12174 PMC4947168

[b49] Lin Y. C. . Single-layer ReS_2_: two-dimensional semiconductor with tunable in-plane anisotropy. ACS Nano 9, 11249–11257 (2015). 26390381 10.1021/acsnano.5b04851

[b50] Xia F., Wang H. & Jia Y. Rediscovering black phosphorus as an anisotropic layered material for optoelectronics and electronics. Nat. Commun. 5, 4458 (2014). 25041752 10.1038/ncomms5458

[b51] Cincotti S. & Rabe J. P. Self-assembled alkane monolayers on MoSe_2_ and MoS_2_. Appl. Phys. Lett. 62, 3531 (1993).

