# Supplementary material for: Periodic potentials in hybrid van der Waals heterostructures formed by supramolecular lattices on graphene
Source: Nat Commun. 2017 Mar 21;8:14767. doi: 10.1038/ncomms14767 (PMC5364416; doi:10.1038/ncomms14767)
Supplement: Supplementary Information — Supplementary Figures, Supplementary Tables, Supplementary Methods, Supplementary Note and Supplementary References. [file ncomms14767-s1.pdf]

Supplementary Figures

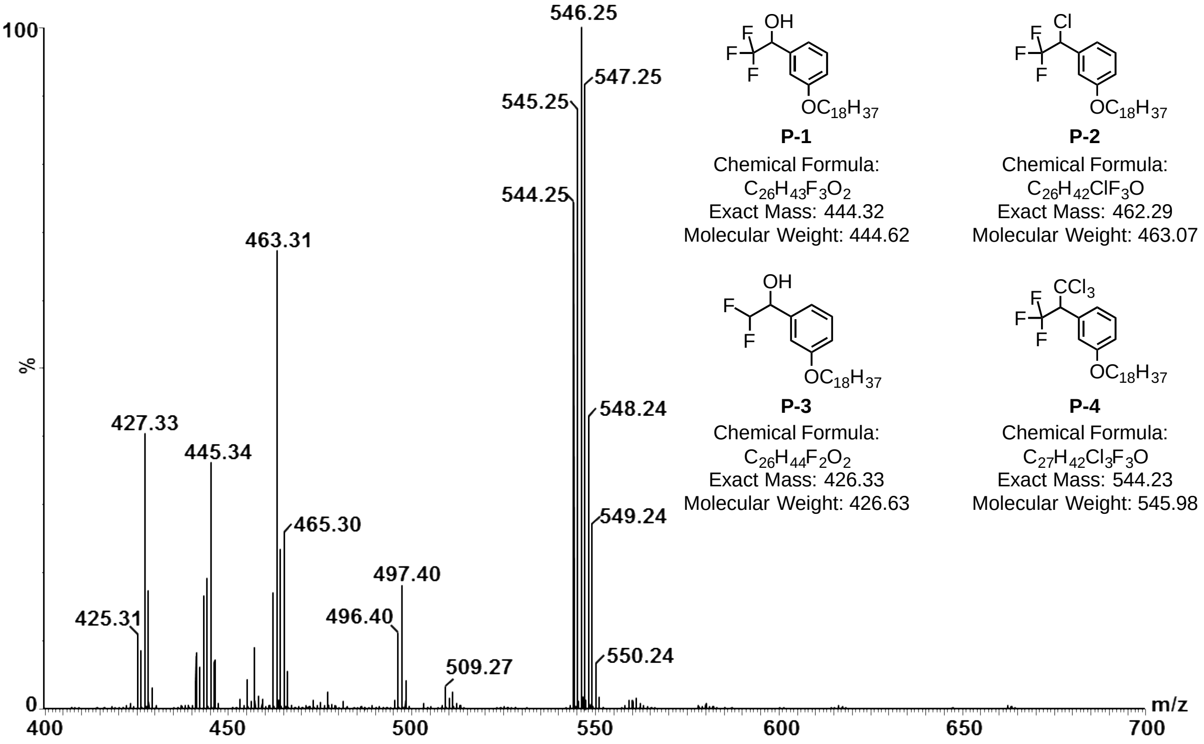

Supplementary Figure 1. Determination of the reaction products in MBB-2: Mass spectrometry. While MBB-1 is the unreacted pure compound, 3-trifluoromethyl-3-(3-octadecyloxyphenyl)diazirine, which is selectively synthesized, MBB-2 is a mixture of reaction products obtained by irradiation of MBB-1 with ultraviolet light at 365 nm in chloroform. The atmospheric solids analysis probe (ASAP) mass spectrometry (MS) analyses of the MBB-2 mixture shown in this Figure indicates the presence of a number of reaction products, including the derivatives called P-1 to P-4, displayed in inset. In the main reaction products, the 3-octadecyloxyphenyl group is maintained while the head is functionalized with another functional group such as hydroxy, chloro, or trichloromethyl.

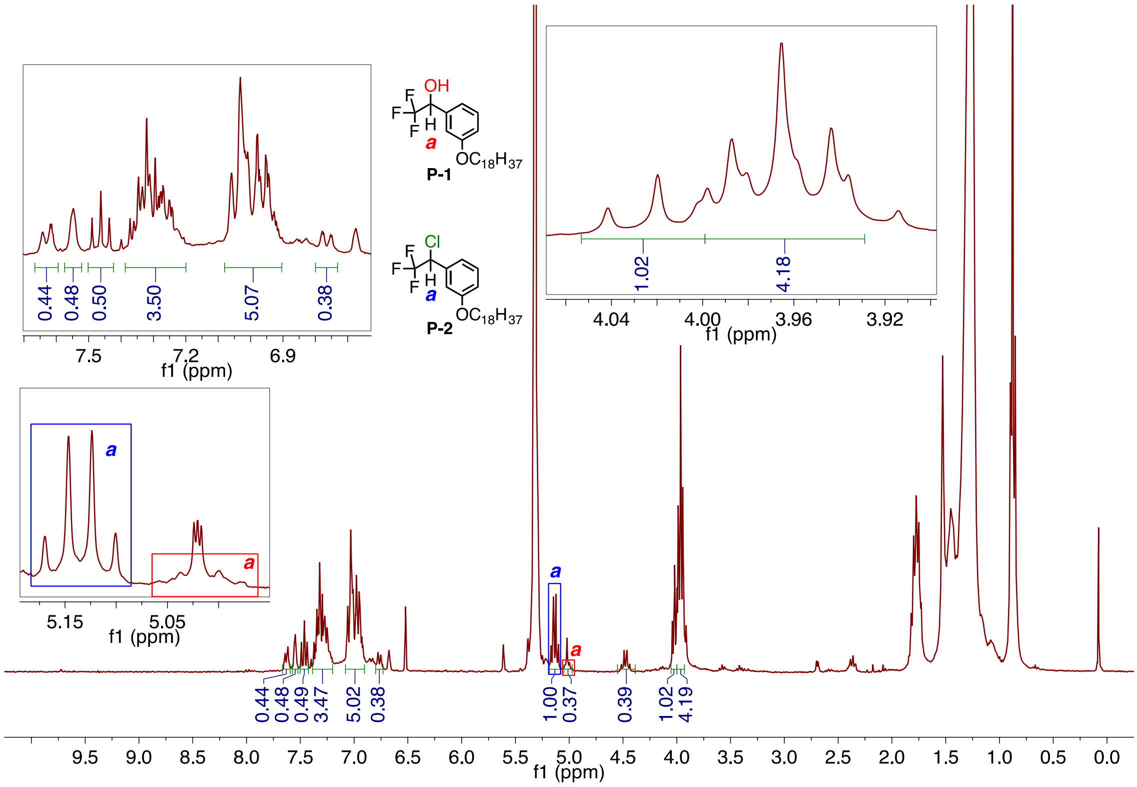

Supplementary Figure 2. Determination of the reaction products in MBB-2: Nuclear Magnetic Resonance spectroscopy. ^{1}H nuclear magnetic resonance (NMR) spectroscopy analysis of MBB-2, i.e., the reaction products from the photolysis of the diazirine derivative in chloroform (300 MHz, CD_2Cl_2). The ^{1}H NMR spectrum shows complex multiplet signals in the aromatic region as well as at 3.9–4.1 ppm, corresponding to the alkoxy protons -OCH_2-, which confirms the presence of different reaction products in agreement with the ASAP MS spectrum in Supplementary Figure 1. Based on the ^{1}H NMR spectra of separately synthesized P-1 and P-2, (Supplementary Figure 3), the quartet peaks centered at 5.01 and 5.13 ppm can be assigned to P-1 and P-2, respectively, as indicated by "*a*" and rectangles in red and blue colors. Considering the ratio of integration of these quartet peaks as well as the total integration of the triplet peaks from -OCH_2- at around 4 ppm as well as the aromatic signals, P-2 can be concluded to be the main component of the MBB-2 mixture. The blue numbers below the spectrum denote the integration of the peaks over the areas indicated by the green bars.

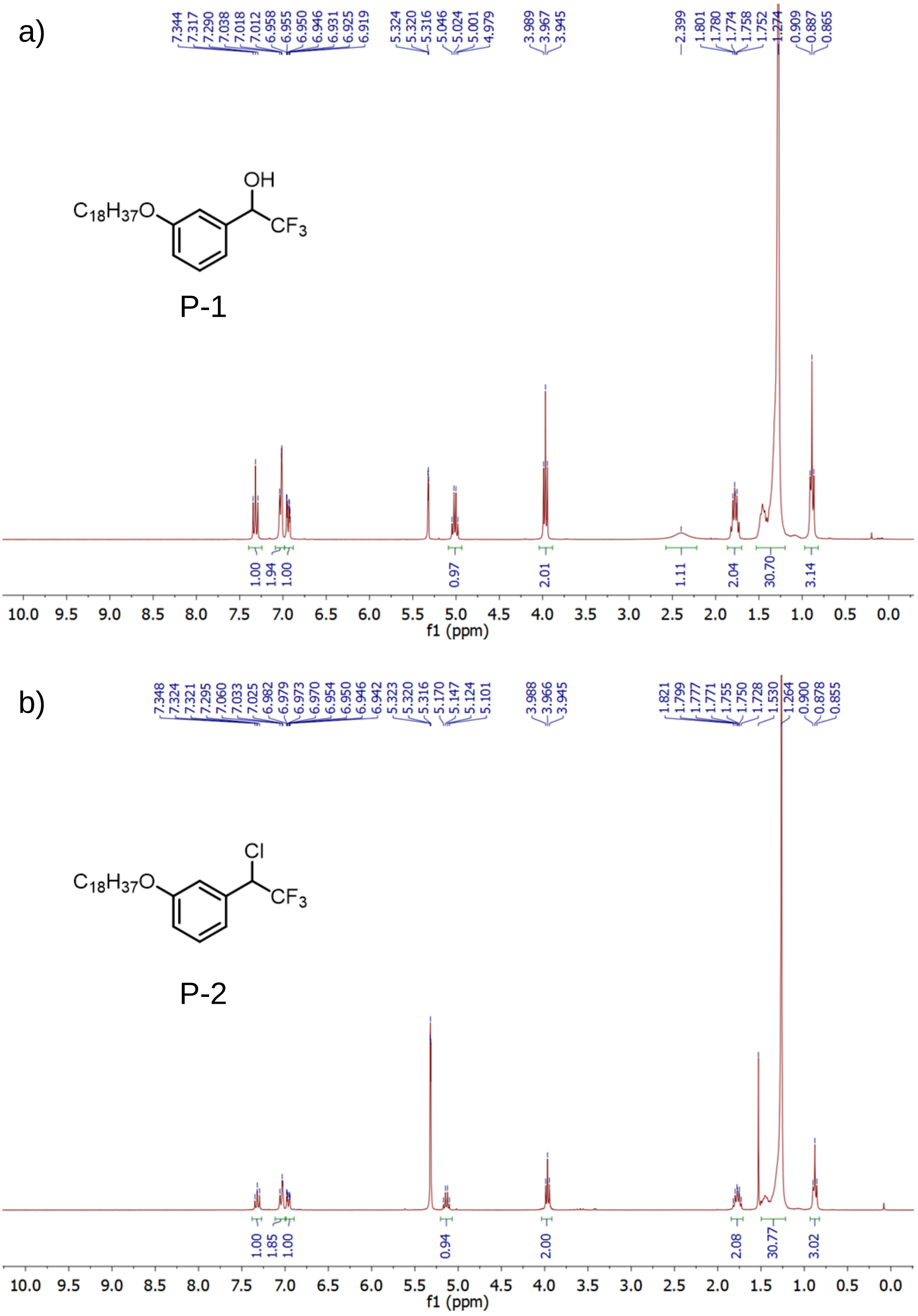

Supplementary Figure 3. ^{1}H NMR spectra of separately synthesized P-1 and P-2. The comparison of the ^{1}H NMR spectra (300 MHz, CD_2Cl_2) of the separately synthesized P-1 (a) and P-2 (b) with that of MBB-2 from the photoreaction (Supplementary Figure 2) confirmed the formation of P-1 and P-2 upon the photolysis of MBB-1 in chloroform (see the caption of Supplementary Figure 2).

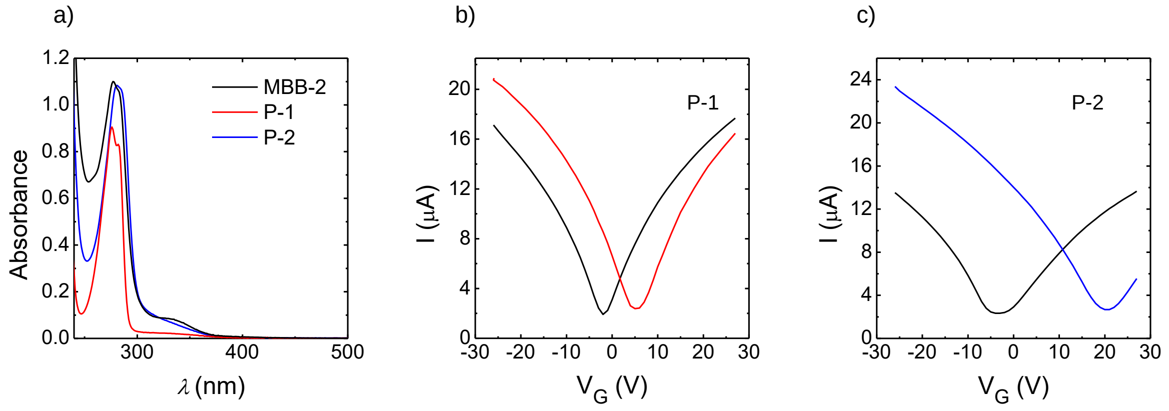

Supplementary Figure 4. Comparison between the mixture MBB-2 and the separately synthesized P-1 and P-2. (a) Comparison of the Ultraviolet (UV)-Visible spectra of the separately synthesized P-1 and P-2 and MBB-2, obtained by irradiating MBB-1 with UV light (concentration of P-1, P-2, MBB-1 2.2 $\times 10^{-3}$ M in chloroform; 2-mm quartz cells; room temperature). The UV-Visible spectra of the irradiated compound MBB-2 is remarkably similar to that of the Cl-modified P-2, confirming that this compound is the main reaction product. (b) Effect of the presence of hydroxyl-modified derivative P-1 on the electrical characteristics of graphene. Black line: pristine graphene device; red line: graphene device covered by a P-1 supramolecular lattice. (c) Effect of the presence of chloro-modified derivative P-2 on the electrical characteristics of graphene. Black line: pristine graphene device; blue line: graphene device covered by a P-2 supramolecular lattice. I is the current flowing through graphene, and V_G is the gate voltage.

As compared to the effect of MBB-2 (Fig. 3b), a significantly lower p-doping was introduced by P-1 (b). Instead, the effect of P-2 (c) is analogous to that of MBB-2. This finding strongly indicates that this compound is the main reaction product on the graphene surface after UV irradiation of the diazirine derivative in $CHCl_3$.

Following this findings, for the simulation of the effect of MBB-2, we assumed that all the molecules on the surface possess the P-2 structure. Although the presence of other reaction products at the surface cannot be fully ruled out, it would be a minor effect which does not change significantly the scientific outcome of this work.

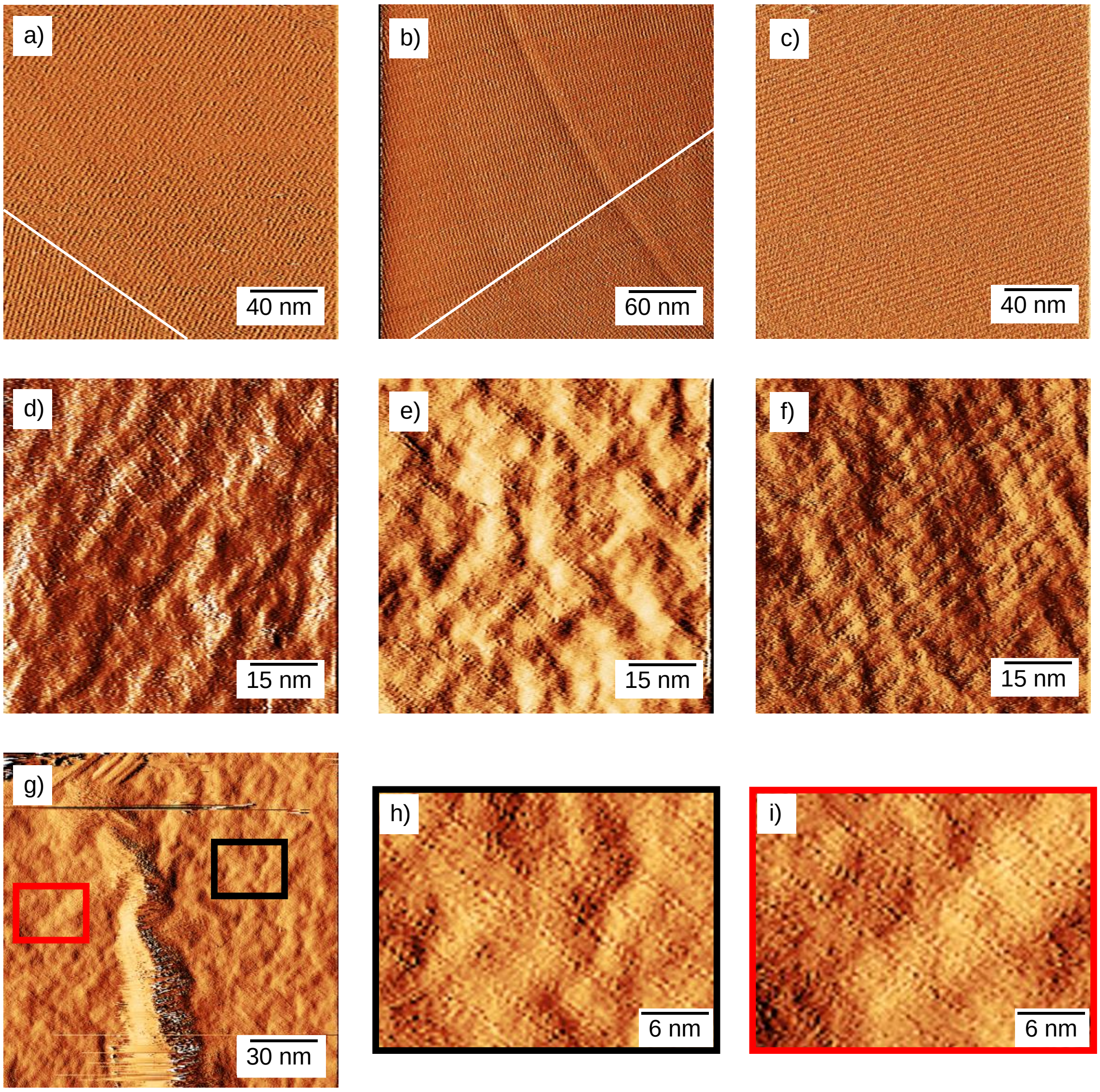

Supplementary Figure 5. Large area STM image of the supramolecular lattices. (a) MBB-1, (b) MBB-2, and (c) MBB-3 on highly oriented pyrolytic graphite. In (b) and (c), two domains with different orientation of the lamellas are visualized, and the domain boundary is highlighted by a white line. (d), (e), (f) STM image of the assembly of (d) MBB-1, (e) MBB-2, and (f) MBB-3 on graphene grown by chemical vapor deposition (CVD) and transferred on a SiO_2 substrate. In the three images, the direction of the lamella is maintained within the whole image. (g) Large area (150 nm × 150 nm) STM image of a single domain of MBB-2 spin-coated on CVD graphene on SiO_2. (h)-(i) Zoom-in of the two regions highlighted in (g), showing that the same direction of the assembly is kept on both sides of the central ripple. Typical tunnelling parameters: tip voltage (V_t) = 400-600 mV, and average tunnelling current (I_t) = 20-50 pA.

We highlight that the images of MBB-2 show a very uniform assembly, suggesting that mostly one compound is lying on the surface. Moreover, the similarity between the unit cells measured for MBB-1 and MBB-2 indicates that the size of the main reaction product is very similar in both cases, ruling out the bigger derivative P-4 as main product on the surface.

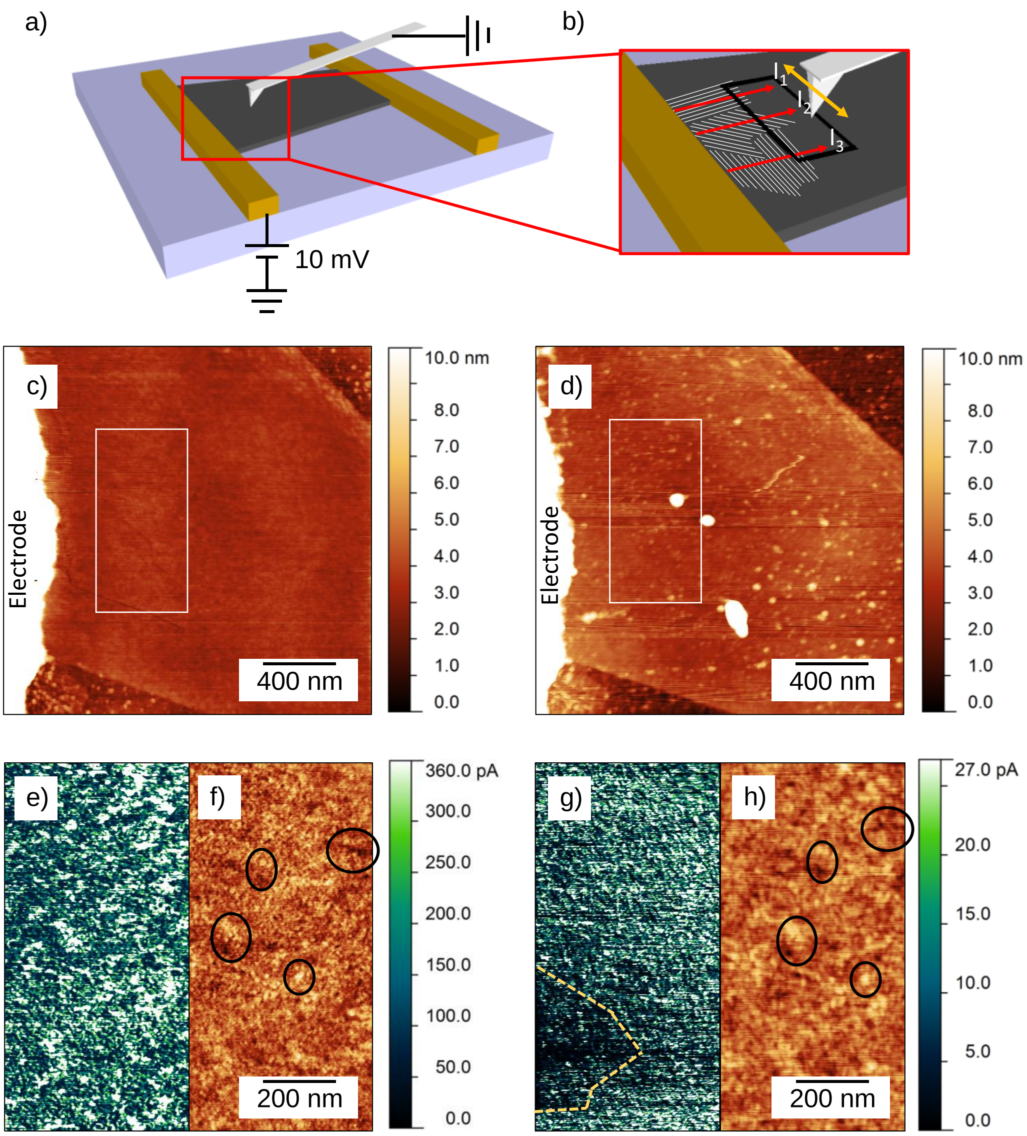

Supplementary Figure 6. Spatially-resolved current distribution. Conductive atomic force microscope (C-AFM) experiments were performed in the same area of the same graphene device before and after the formation of a supramolecular Lattice (SL) of MBB-1**.** (a) Schematic of the measurement. A conductive tip is scanned over the sample while a bias $V = 10$ mV is applied between the tip and a gold electrode, allowing to simultaneously measure morphology and current. (b) Cartoon of the effect of domain with different lamella orientation. The white lines represent SL domains with different lamella orientation while the black rectangle represents the scan area. I_1, I_2 and I_3 are the currents flowing across the SL/graphene area covered by SL domains with different lamella orientation. Owing to the anisotropy on graphene conductivity induced by the presence of the SL, the current reaching the tip depends on the local lamella orientation between electrode and tip. In a current map, this effect would be recorded as a spatial modulation of the current, with a length-scale comparable to that of single SL domains ($I_1 \neq I_2 \neq I_3$). (c, d) Morphology images of the graphene device at relatively large scale (2×2 μm^2) before (c) and after (d) the SL formation. The pristine graphene is extremely clean (c). After the SL formation, we found that the same area was covered

with a molecular adlayer (d). The morphology of such layer is different from that of pristine graphene because a few protrusions, separated by flat regions, could be imaged by AFM. Such flat regions as areas in which graphene is covered strictly by one monolayer, and the protrusions as 3D molecular aggregates. We note that the electrical contacts do not impact significantly the order within the molecular layer; at the least, no evident material accumulation/depletion close to the contact is encountered. (e-h) Current and morphology maps measured before (e and f) and after (g and h) the SL formation, recorded in the smaller region of the device in the areas highlighted in (c) and (d), at a distance approximately 200 nm from the electrode. Such distance is shorter than the typical size of a SL single domain, so it can be assumed that only one or at most two SL domains are comprised between the electrode and the closer side of the image. By comparing morphology images recorded before and after the SL formation (f and h), one can univocally recognize the same surface features (some of which are indicated by the black circles), confirming that the images were recorded in the very same area. The lamella orientation could not be resolved, most probably since the 3.8-nm stacking is below the resolution limit of the C-AFM.

Noteworthy, the current images measured before and after the SL are significantly different. The current map measured for the pristine device (e) displays a short-range modulation with a typical length scale of a few tens of nm^2, which follows the graphene roughness, as can be noticed by comparing the current map (e) and morphology image (f), recorded simultaneously. Such short-range current modulation can be ascribed to the tip-graphene contact that locally varies owing to graphene roughness that affects the current injection locally. The current map measured after the SL formation (g) displays two main differences as compared to the pristine case: (1) the current averaged over the whole image is more than one order of magnitude lower (I_{after} = 14 pA vs I_{before} = 250 pA) and (2) the current is inhomogeneous on a length scale of a few-hundreds nm^2. The lower average current can be explained considering that the presence of the insulating molecular layer on the surface of graphene increases the overall resistance of the system, and that after the SL formation we recorded the image applying a lower force between the tip and the sample (2 nN before and 1.5 nN after the SL formation), in order to minimize the removal of molecules by the tip. More interestingly, we found that the presence of the SL introduces inhomogeneities in the conductance of graphene. In particular, we observe a region of a few hundred nm^2 with markedly lower current, delimited by the yellow dotted guide to the eye in panel (g). The average current measured within this region is one third of the average current outside of it (5 pA vs. 15 pA). In (h) there are no features which could explain the lower current measured in such region. A very similar current map was obtained by scanning the same region with the fast scan direction either parallel or perpendicular to the electrical contact, ruling out possible artefacts related to tip deterioration during the imaging. The size of the low-conductance region (few hundred nm^2) roughly matches that of single crystalline domains of the SL. As explained above, this experimental evidence supports the presence of anisotropic conductance within graphene areas covered by SLs with random local lamella orientation.

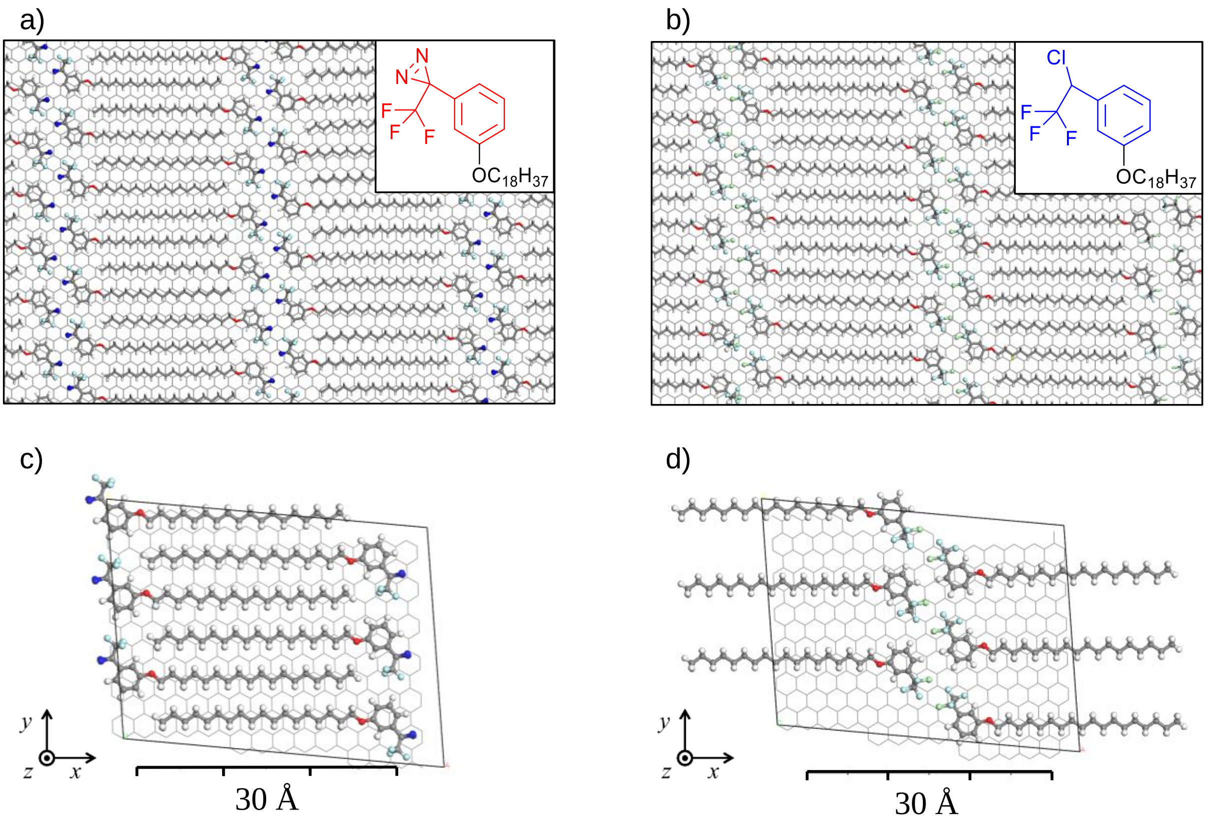

Supplementary Figure 7. Assemblies and unit cells predicted *via* molecular dynamics simulations. (a,b) Predicted assemblies for MBB-1 (a) and MBB-2 (b). Insets: molecular structures of MBB-1 and MBB-2. (c,d) Unit cells for MBB-1 (c) and MBB-2 (d).

The force field molecular dynamics simulations show that both the MBB-1 and MBB-2 are packed in a lamellar structure with head-to-head configuration. For both molecular adlayers, the smallest repeating motives (c,d) of the supramolecular lattice have been carefully extracted accounting for the atomic positions of the diazirine derivatives relative to the carbon atoms of the underlying graphene. In these unit cells, six molecules (i.e., three dimers) are required for a reasonable commensuration of the molecular adlayers with the graphene surface. Thus, the calculated lattice parameters of the unit cell are: $a \approx 3.7$ nm, $b \approx 2.8$ nm (area ≈ 10.2 nm^2) and $\alpha \approx 81°$. We note that the calculated unit cell parameters were found for both MBB-1 and MBB-2, and are in very good agreement with the experimental data (see main text). The energy minimization of the supramolecular lattice/graphene systems was performed in vacuum, under periodic boundary condition (PBC), and carried out using the Conjugate-Gradient algorithm, until the RMS force was less than 5×10^{-3} kcal mol^{-1} Å with energy convergence of 1×10^{-4} kcal mol^{-1} between steps. Series of 25-ps-quenched dynamics (T = 50,100, …, 300 K, quench frequency = 1 ps) for which different temperatures were used so that the energy between quenched dynamics would no longer decrease. The molecular dynamics simulations were conducted in the NVT ensemble, using the Nosé thermostat.

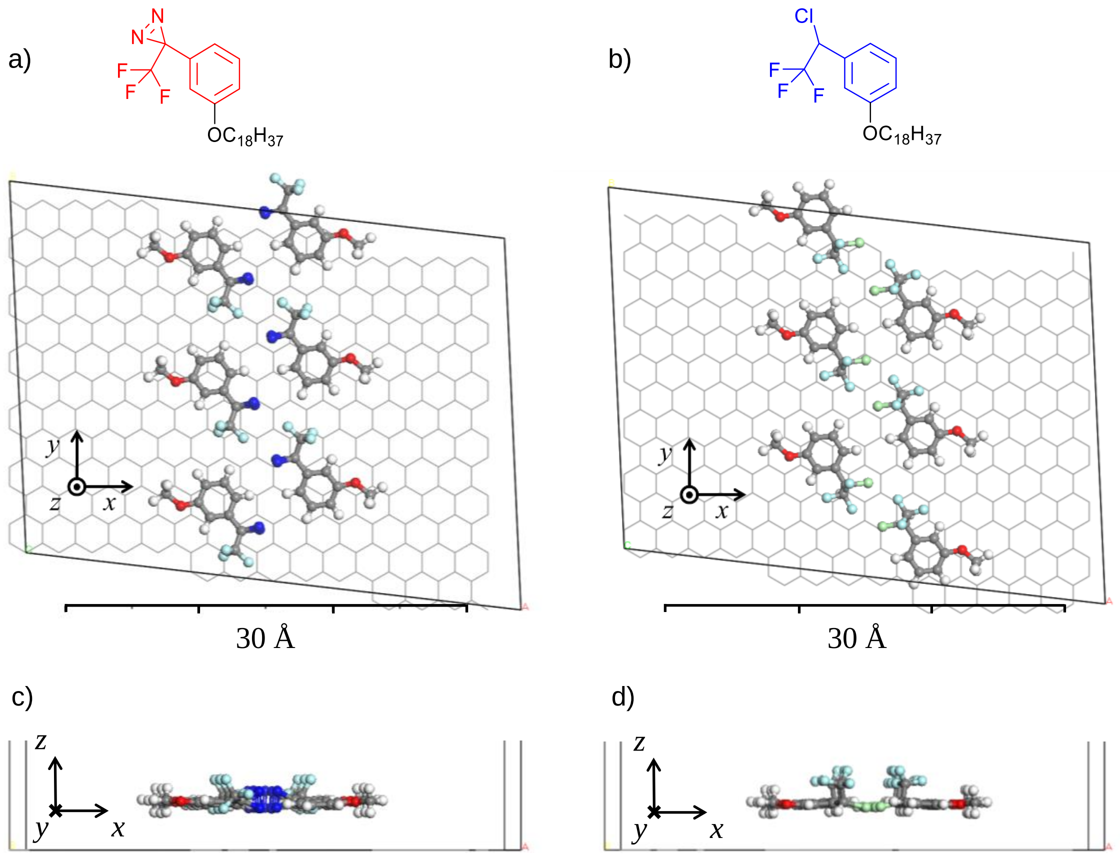

Supplementary Figure 8. Unit cells used in the density functional theory calculations. (a) Top view and (c) side view of the unit cell used in the density functional theory (DFT) calculations for MBB-1. (b) Top view and (d) side view of the unit cell used in the DFT calculations for MBB-1. Using these unit cells, DFT calculations were performed to evaluate the charge density redistribution as well as the influence of the electrostatic potential induced by the supramolecular lattices on the work function of graphene. The atomic positions have been optimized at the molecular mechanics/molecular dynamics level, while no optimization of the unit cell has been performed at the DFT level. In order to considerably reduce the computation time, the linear alkyl chains were removed, i.e., $C_{21}H_{43}$ groups were substituted by CH_3 groups. Indeed, the saturated chains should not contribute to the doping of graphene. Thus, the unit cells consist in a graphene layer of 388 carbon atoms and 6 (MBB-1 or MBB-2) moieties on top of it.

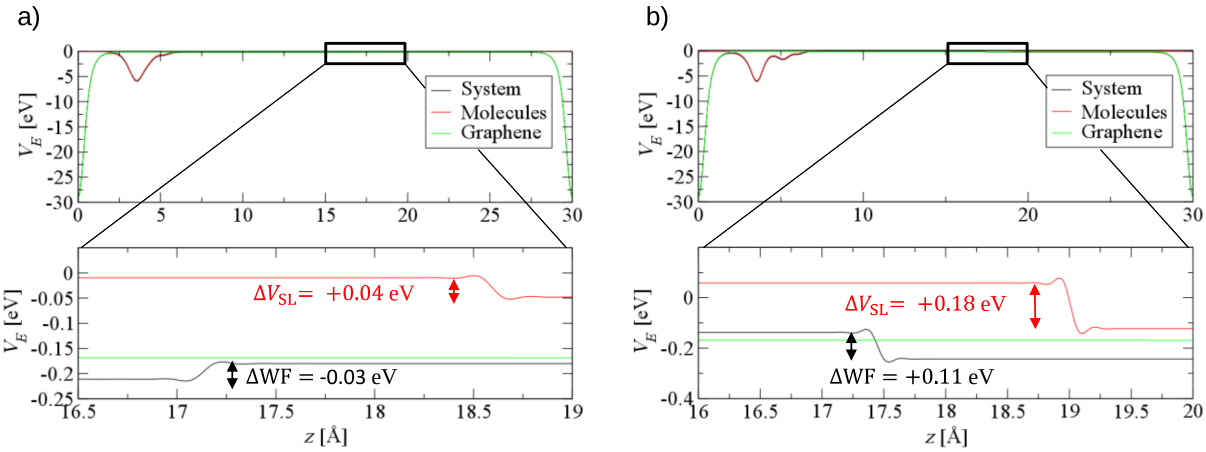

Supplementary Figure 9. Potential profile at the graphene/supramolecular lattice interface from density functional theory. (a) Profile of the potential V_E across the graphene/MBB-1 interface; (b) Potential profile across graphene/MBB-2 interface (*z*-direction normal to the graphene surface). (c), (d) Zoom on the vacuum level shift (far from the interface). Black line: potential of the entire system composed of graphene + supramolecular lattice (SL), related to the change in work function ΔWF. Red line: potential of the isolated SL, the variation of which will be called ΔV_{SL}. Green line: potential related to the charge transfer contribution to graphene ΔV_E.

The plane-averaged electrostatic potential *V*(z) was calculated numerically from the self-consistent SIESTA potential *V*(*x*,*y*,*z*) using the macroscopic average technique developed by Baldereschi et al. [1]

Following the methodology described in Supplementary Methods, to distinguish the contributions to the work function shift ΔWF (intrinsic dipole moment of the SL ΔV_{SL} and charge transfer ΔV_E), we analysed the potential profile of the full supramolecular lattice (SL)-graphene system and the isolated SL. In the case of MBB-1, the decomposition shows that the contribution of the SL (ΔV_{SL}= +0.04 eV) is compensated by the charge transfer term (ΔV_E = -0.07 eV), leading to a relatively weak decrease of work function (ΔWF = -0.03 eV). In the case of MBB-2, ΔV_{SL} and ΔV_E are opposite in sign, meaning their effects partly cancel out, yet the net effect is a WF shift of approximately 0.10 eV. Such a work function shift is indicative of a p-type doping, which is in line with experimental observations. Interestingly, it is found that the energy term associated to the charge transfer is identical in the two cases (approximately ΔV_E = -0.07 eV). This indicates that ΔWF for MBB-2 mainly results from the modification of the intrinsic dipole moment of the SLs.

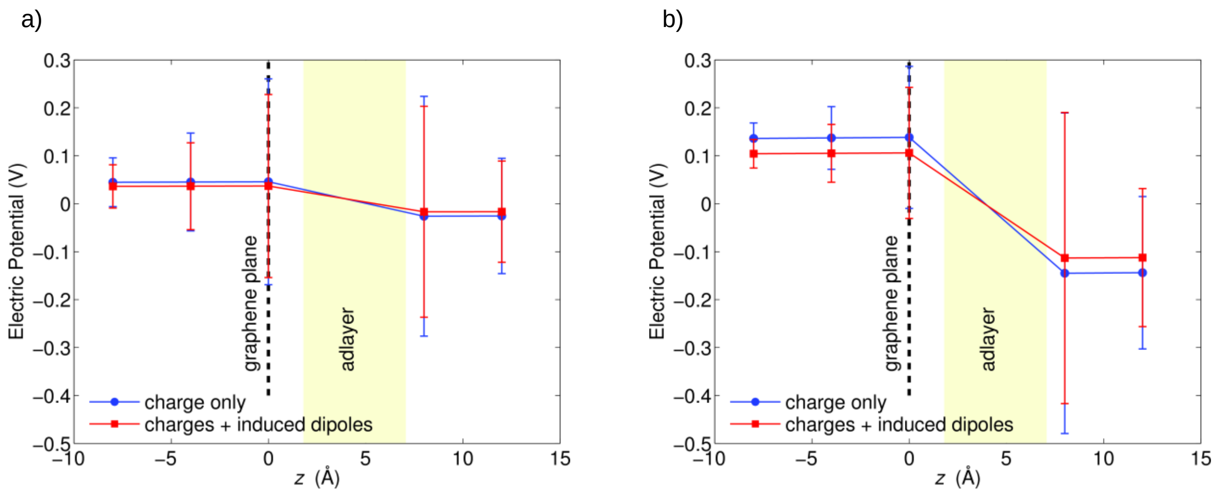

Supplementary Figure 10. **Potential profile at the graphene/supramolecular lattice interface from microelectrostatic calculations.** (a) Calculations for MBB-1 and (b) for MBB-2 supramolecular lattices adsorbed on graphene. As compared to Supplementary Figure 9, the potential profile shown in this picture is analogous to the contribution in the work function shift due to the presence of the molecular electrical dipoles ΔV_{SL}.

Symbols and error bars show the average and standard deviation of the potential in planes at different distances from the $z = 0$ graphene plane. Circles and squares show the potential profile obtained with the gas-phase molecular charge distribution, approximated by atomic charges from electrostatic potential fitting (charge only), and accounting for molecular polarizability (charges + induced dipoles). The potential step across the plane reduces from 0.28 eV to 0.22 eV when the polarizability of the MBB-2 adlayer is accounted for, whereas the change is negligible when polarizability of the MBB-1 is considered.

The presence of the organic adlayer induces a change in the electrostatic potential when crossing the graphene plane, and the potential step corresponding to MBB-2 is four times larger than that of MBB-1. In particular, the polar CF_3 groups pointing their more electronegative fluorine atoms away from the surface result in an electrostatic potential that is shifted to large negative values on top of the aromatic cores, while smaller positive values are computed on top of the alkyl chains. Overall, the microelectrostatic (ME) calculations indicate a shift in the potential consistent with p-doping of the graphene layer, in quantitative agreement with the DFT results (ΔV_{SL} = 0.18 eV from DFT against approximately 0.2 eV from ME, calculated for MBB-2 (b).

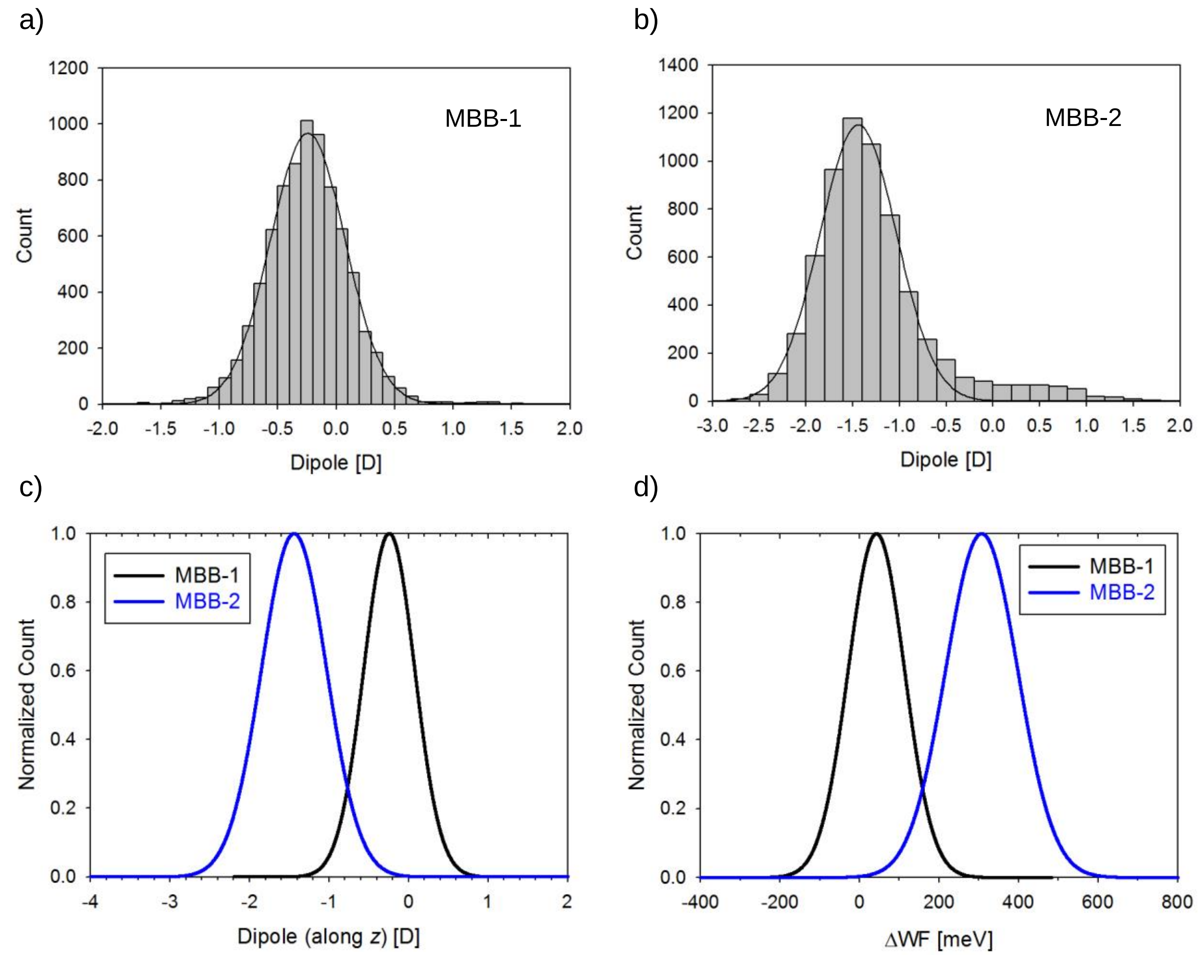

Supplementary Figure 11. Thermal distribution of molecular dipoles within the assembly and correspondent distribution of work function shift. (a,b) Thermally-smeared distribution of electrical dipoles calculated by molecular dynamics (MD) for MBB-1 (a) and MBB-2 (b). (c) Comparison of the fits to the electrical dipole distribution for MBB-1 and MBB-2, as extracted by (a) and (b). (d) Corresponding work function distribution for MBB-1 and MBB-2.

At room temperature, the supramolecular lattices undergo thermal vibrations and, therefore, the molecules can explore multiple configurations at the graphene surface. In turn, the different configurations are characterized by different orientation of molecular dipoles, which determine different work function shifts (ΔWF). To build a distribution of the molecular dipoles, we first performed MD simulations on the SL-graphene systems previously calculated (from which the unit cells were extracted). A molecular dynamics simulation of 100 ps was then performed and the electric dipoles of the molecules in the supramolecular lattice were recorded every 1 ps (100 frames). Next, we analyzed the correlation between the direction of the dipole moment and the geometry of the diazirine moiety on the graphene surface. For the MBB-1 derivative, we found a single population in which the molecule is flat on the graphene surface with the CF_3 group oriented in plane (Figure 3c and 3d in the main text). As shown in (a), the MD-calculated electric dipoles follow a Normal

distribution, ranging from -1.5 to 1 D, with an average value and standard deviation of -0.2 and 0.3 D, respectively. In the case of MBB-2, a single population of electric dipole is also observed, yet now the Normal distribution is centered at -1.4 D with a standard deviation of 0.4 D (b). The geometry associated to this dipole distribution is shown in Figure 3d in the main text. As detailed in Supplementary Methods, we calculated a distribution of ΔWF (including a fixed ΔV_E contribution) for each dipole configuration for MBB-1 and MBB-2. The results reported in (d) have been renormalized per molecule (by dividing the surface by the number of molecules in the unit cell). The average and the standard deviation of the ΔWF distribution are 0.04 eV and 0.071 eV (0.31 eV and 0.09 eV) for the MBB-1 (MBB-2) derivative, respectively. Therefore, when the thermal vibrations are taken into account the ΔWF for MBB-2 increases to 0.31 eV from the 0.10 eV calculated at the density functional theory (DFT) level, indicating a strong p-doping in line with the experimental findings. Instead, the MD calculated ΔWF associated to MBB-1 has a sign opposite to that calculated at the DFT level. Yet, the main effect is weak in both cases, with differences arising from the neglect of depolarization effects in the MD simulations or of thermal effects in the DFT calculations.

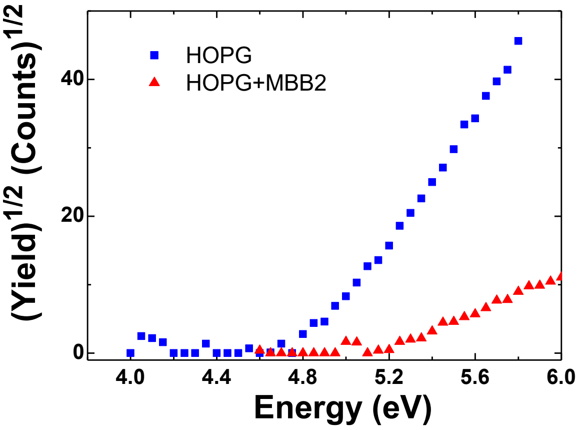

Supplementary Figure 12. Measurement of the work function shift through photoelectron spectroscopy in air. Representative measurements of the work function (identified by the intercept of the linear fitting of the curve with the Energy axis) for a freshly-cleaved highly oriented pyrolytic graphite HOPG sample and for the same surface after spin-coating MBB-2. The measurements were carried out by photoelectron spectroscopy in air (PESA) with an AC-2 Photoelectron Spectrometer (from Riken-Keiki Co.). The ultraviolet (UV) incident light power used for the measurements was 200 nW with a counting time of 10 seconds per point. As a substrate, we employed a 10 mm × 10 mm HOPG substrate, which can be cleaved to obtain clean surfaces. First, we took photoemission spectra in three different region (2 mm × 2 mm) of a freshly cleaved HOPG sample. Subsequently, we spin-coated MBB-2 on the same surface and repeat the measurements by probing different regions. A representative measurement before and after the SL formation is shown in Supplementary Figure 15.

Averaging over ten samples, we obtained WF=4.85 ± 0.05 eV for the freshly cleaved HOPG surface and WF= 5.25 ± 0.15 eV for the HOPG surface covered by MBB-2. The work function shift ΔWF = 0.4 ± 0.2 eV is in good agreement with the theoretical calculations. We also highlight that a ΔWF = 0.3 eV shift in the graphene work function was found to correspond to a change in the induced charge density $\Delta p = 5 \cdot 10^{12}$ cm^{-2},[2–4] in excellent agreement with the experimental result for MBB-2 (see the main text).

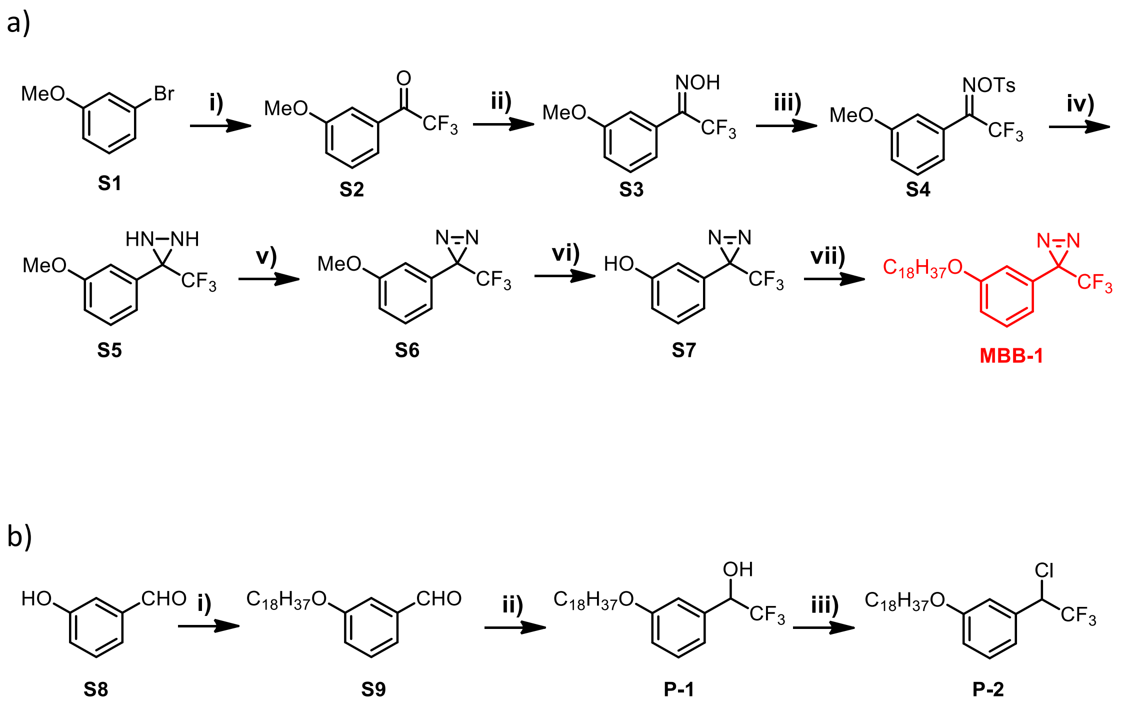

Supplementary Figure 13. Synthesis of MBB-1, P-1 and P-2. (a) Synthetic route to the molecular building block MBB-1 with a long alkoxy chain. The diazirine precursor 3-(3-(trifluoromethyl)-3*H*-diazirin-3-yl)phenol (S7) was synthesized according to the literature.[18] Reagents and conditions: i) *n*-BuLi, Et_2NCOCF_3, THF, –78 °C, 3 h; ii) NH_2OH-HCl, EtOH, 70 °C, 4 h; iii) TsCl, NEt_3, DMAP, DCM, RT, 24 h; iv) NH_3, DCM, –78 °C, 12 h; v) Ag_2O, Et_2O, RT, 20 h; vi) BBr_3, DCM, RT, 12 h; vii) $C_{18}H_{37}Br$, K_2CO_3, acetone, 60 °C, 65%. THF: tetrahydrofuran; Ts: *p*-toluenesulfonyl; DMAP: 4-dimethylaminopyridine; DCM: dichloromethane. (b) Synthetic route to P-1 and P-2**.** Reagents and conditions: i) $C_{18}H_{37}Br$, K_2CO_3, acetone, 60 °C, 89%; ii) $TMSCF_3$, K_2CO_3, DMF, RT, 12 h; then HCl (1.0 M, aq), RT, 1 h, 85%; iii) $SOCl_2$, pyridine, DMF (cat.), 80 °C, 63%. DMF: dimethylformamide. The detailed synthetic procedures and characterizations are described in Supplementary Note 1.

Supplementary Tables

MBB-1				
Image Size (nm^2)	# images	# images with single domain	# images with two domains	# images with three domains
60 × 60	6	5	1	0
80 × 80	4	2	2	0
90 × 90	1	0	1	0
100 × 100	2	1	1	0
200 × 200	1	1	0	0
300 × 300	1	0	1	0
**MBB-2**				
Image Size (nm^2)	# images	# images with single domain	# images with two domains	# images with three domains
60 × 60	5	3	2	0
80 × 80	1	0	1	0
90 × 90	1	0	1	0
100 × 100	3	2	1	0
150 × 150	2	2	0	0
200 × 200	3	0	2	1
**MBB-3**				
Image Size (nm^2)	# images	# images with single domain	# images with two domains	# images with three domains
60 × 60	4	3	1	0
100 × 100	2	1	1	0
200 × 200	1	1	0	0

Supplementary Table 1. Summary of large-area scanning tunnelling microscopy images recorded for MBB-1, MBB-2 and MBB-3. For each molecular building block (MBB), we recorded at least 80 STM images considering both highly oriented pyrolytic graphite and CVD graphene on SiO_2. For relatively small images (< 40 nm × 40 nm), we always observed a single domain in the image – corresponding to lamellas oriented in the same direction all over the image. Therefore, only relatively large images (> 60 nm × 60 nm) are relevant for an estimation of the average domain size. This table provides a detailed analysis of these images, highlighting the number of domains encountered on each large-scale image. Limiting the discussion to the relevant data (images size > 60 nm × 60 nm), we have measured 15 images for both MBB-1 and MBB-2, and 7 for MBB-3.The images were recorded in different STM sessions, on different samples (prepared in the same way). In the case of images recorded one after the other in the same spot, only the first one was taken into

account. In all large-area images, either a single domain or two domains separated by a domain boundary are imaged (additional large-area STM images are shown in Supplementary Fig. 7). An entire domain would never be fully included into a single image, even on the largest scale. Hence, we conclude that single domains are typically wider than our maximum image size – typically 200 nm × 200 nm. The fact that a domain boundary is often imaged brings us to conclude that a reasonable value for the typical domain size is 300 × 300 nm.

	μ_x [D]	μ_y [D]	μ_z [D]	ΔV_{SL} [eV]	ΔV_E [eV]	ΔWF [eV]	Doping
MBB-1	0.01	0.00	-0.17	+0.04	-0.07	-0.03	n
MBB-2	0.03	0.01	-0.82	+0.18	-0.08	+0.10	p

Supplementary Table 2. Molecular dipoles and variation of the work function of graphene upon physisorption of different supramolecular lattices. The electric dipole per molecule along the three direction μ_x, μ_y, μ_z are expressed in Debye, while the contribution to the electrostatic potential associated to the intrinsic dipole moments of self-assembled monolayers (ΔV_{SL}) and to charge transfer (ΔV_E) as well as the resulting work function shift (ΔWF) are in electronvolt. The resulting doping type is also indicated. As detailed in Supplementary Methods, the molecular dipoles have been calculated by molecular dynamics simulations, while the variations in the surface potential by Density Functional Theory. Since ΔV_E is analogous in both cases, the difference in ΔWF can be ascribed mostly to the different vertical dipoles μ_z.

F is a strongly electron-withdrawing atom, so one would expect F-rich molecules to induce strong charge transfer. Indeed, molecules such F_2-HCNQ[5] and F_4-TCNQ[2] are known to be efficient dopants for graphene. All compounds encompassed within our study comprise a -CF_3 group which could be thought of as a source of electron extraction from graphene as well. However, this was not found to be the case in our study, as evidenced by the relatively weak ΔV_E for MBB-1 and MBB-2. Actually, in order to obtain efficient charge transfer, the molecular levels should lie close to the Fermi level of graphene. When that is the case, an electron flow can take place from or to graphene to reach equilibrium, possibly mediated by partial molecule-graphene hybridization and the formation of interfacial states acting as scattering or trapping centers[6]. The fact that fluorinated molecules are often used as dopants is due to the fact that very often in conjugated molecules the presence of withdrawing electron groups (F atoms) lowers the LUMO and brings it closer to the Fermi level of graphene. Instead, in our system, the LUMO level of the molecule lies far in energy from the Fermi level of graphene, thus preventing efficient charge transfer. Indeed, we calculated the frontier orbitals of a methoxy-trifluoromethyl-phenyl-diazirine in chloroform at the B3LYP level (see Supplementary Methods). The LUMO lies at -2.34 eV, which is more than 2 eV higher than the Fermi level of graphene (-4.6 eV).[7] Moreover, it has been reported recently that the effect of dielectric fluoro polymers on the graphene characteristics is very similar to what is reported here for MBB-1: minor overall doping, with an increase in the graphene mobility.[8] In conclusion, it is not generally true that F-rich molecules withdraw electrons, and in particular MBB-1 and MBB-2 introduce minor charge transfer. Instead, their doping effect measured is mediated by the presence of aligned out-of-plane molecular dipoles, as shown by our calculations.

Supplementary Methods

Methodology for the modeling. The formation of a MBB-1 supramolecular lattice (SL) is investigated by molecular mechanics/dynamics (MM/MD) simulations. The change in the work function (WF) due to the photo-reaction of the diazirine molecules is analyzed using density functional theory (DFT) calculations. Following the above discussion, for the simulation of the effect of MBB-2, we assumed that all the molecules on the surface possess the P-2 structure. The doping is evaluated in terms of work function shift (ΔWF) of graphene in the presence of SL of MBBs. The procedure involves three successive steps: i) force field parameterization based on quantum-chemistry calculations; ii) MD simulations of the supramolecular 2D architectures and extraction of the unit cell from the optimized SL; and iii) DFT electronic structure calculations on the optimized unit cell. Note that the combination of (ii) and (iii) allows converting the MD distribution of the molecular conformation and electric dipole of the individual diazirine molecules into WF distributions.

Force field parameterization. An all-atom description was used for the individual molecules, i.e., all hydrogen atoms were taken into account. For the aliphatic chains of the molecules, the COMPASS force field (FF) was used for all the atomic charges, except for the first –CH_2– unit close to the MC functional group. For the latter, the atom types were described at the quantum chemistry level, with atomic charges calculated from the electrostatic potential (ESP charges)[9] on the basis of MP2/cc-pvdz optimized geometry, as implemented in the Gaussian09 package.[10i] Then chemically equivalent atoms were set with equal (averaged) charges. To describe the phenyl-CF_3 torsional potential at the Molecular Mechanics level of theory, the Dreiding force-field was re-parameterized against reference MP2/cc-pvdz torsional potentials. All MM/MD calculations were performed with the Materials Studio 6.0 package using our fine-tuned Dreiding force-field.

Molecular dynamics simulation details and unit cell extraction. Prior to the construction of the SLs, preliminary MD calculations were run in order to determine the relative stability of different orientations of the molecules with respect to the graphene layer, and between molecules (the relative orientations and shifts of the functional groups and alkyl chains, the inter-digitation of the alkyl chains, etc.). The SLs of MBB-1 or MBB-2 were built atop the graphene surface, which consists in a layer of 14400 carbon atoms (222 × 170 $Å^2$); the graphene layer was spatially frozen, as its geometry is expected to be weakly perturbed upon physisorption of the molecules. Four stacks of 16 (i.e., 64) molecules were initially placed at ~3 Å from the graphene surface (alkyl chains parallel to the zigzag main axis) with adjacent diazirine derivatives in antiparallel configuration, assuming an interdigitation pattern for the alkyl chains with inter-distance of 4.3 Å. Then geometric optimization of the supramolecular 2D architecture of the MBB-1 or MBB-2 was performed (see Supplementary Fig. 9).

DFT calculations and estimation of the doping. We performed DFT calculations under periodic boundary conditions using the SIESTA software package[11] to evaluate the charge density redistribution as well as the influence of the electrostatic potential induced by the SL on the WF of

graphene. The Perdew-Burke-Ernzerhof functional (GGA) has been chosen for the description of the exchange-correlation as it was shown to correctly describe interfacial electronic structure and charge transfer between graphene and various donor/acceptor physisorbed on graphene (and other) surfaces.[12,13] A DZP basis set is used to describe the valence electrons and Troullier-Martins pseudopotentials are used to describe the core electrons. The mesh cutoff was set to 250 Ry and we used a k-sampling of 9×9×1 in the Monkhorst-Pack scheme. This setup gave a WF of 4.6 eV for the pristine graphene layer, which is in good agreement with experimental data.[7]

The work function of a substrate WF is defined as the energy required to extract an electron from the bulk to the vacuum level:

$$\mathrm{WF} = V_{\infty} - E_{\mathrm{F}} \tag{1}$$

where V_{∞} is the electrostatic potential in vacuum and E_F is the Fermi level of graphene. The contribution of the interface potential to the work function shift can be estimated via the charge density difference $\Delta\rho$ at the interface:

$$\Delta\rho(z) = \rho_{\mathrm{sys}} - (\rho_{\mathrm{SL}} + \rho_{\mathrm{graphene}}) \tag{2}$$

where ρ_{sys}, ρ_{SL}, and $\rho_{graphene}$ are the charge density of the whole system (interface), the SL and the graphene layer, respectively. The electrostatic potential ΔV_E associated to the charge density difference at the interface $\Delta\rho$ is obtained by a numerical integration of the Poisson equation:

$$\frac{\mathrm{d}^2 V_{\mathrm{E}}}{\mathrm{d}z^2} = -\frac{\Delta\rho}{\varepsilon_0} \tag{3}$$

The work function shift ΔWF can be expressed as a combination of two main contributions:

$$\Delta\mathrm{WF} = \Delta V_{\mathrm{SL}} + \phi = \Delta V_{\mathrm{SL}} + \Delta V_{\mathrm{E}} + \Delta V_{\mathrm{graphene}} \tag{4}$$

where ΔV_{SL} is a shift of the electrostatic potential induced by the intrinsic dipole moment of the SL, and ϕ is the potential change upon adsorption of the SL on graphene. The latter can be decomposed in two terms, namely the geometric rearrangement of the substrate $\Delta V_{graphene}$ and the electronic reorganization or charge transfer at the interface ΔV_E.[14]

Upon physisorption of the SL on graphene, no geometric restructuration of the carbon atoms occurs ($\Delta V_{graphene} = 0$). Therefore, the change in work function, ΔWF, can be expressed in terms of the local electrostatic potential associated to the charge density redistribution ΔV_E at the interface, and the intrinsic dipole moment of the SL, ΔV_{SL}. By calculating the potential profile across the molecules

while keeping the geometry fixed, the SL contribution can be computed. The charge density redistribution can then be calculated by subtracting ΔV_{SL} from ΔWF. Besides, the intrinsic dipole moment of the SL can be calculated knowing the electric dipole of the molecules in the SL. Indeed, in the Helmholtz model, the molecular contribution is directly proportional to the electric dipole of the molecules along the axis normal to the graphene surface, μ:

$$\Delta V_{\mathrm{SL}} = \frac{-\mu e}{\varepsilon_0 S} \tag{5}$$

where e is the elementary charge, ε_0 the vacuum permittivity, and S the surface area of the unit cell.

Microelectrostatic Calculations. To gain further insight on the WF shift due to the molecular adsorbate we performed classical microelectrostatic (ME) calculations.[15] An accurate polarizable model based on atomic ESP charges and polarizabilities has been built from DFT calculations. The molecular polarizability tensor has principal components α_1=71, α_2=45 and α_3=37 $Å^3$ at the B3LYP/6-311G** level, where the first principal axis is approximately oriented along the alkyl chain, and the third one points normal to the adlayer plane. Induced dipoles at all atoms of the molecular adlayer have been determined self consistently, i.e. fully accounting for their mutual interactions. Periodic boundary conditions are taken into account by computing the interact

tions between the 2D graphene-adlayer cell and its periodic replica within a 50 nm cut-off radius, ensuring converged electrostatic sums.[16]

Supplementary Note 1

Synthetic procedures and characterizations.

3-(3-(octadecyloxy)phenyl)-3-(trifluoromethyl)-3*H*-diazirine (MBB-1). To a solution of 3-(3-(trifluoromethyl)-3H-diazirin-3-yl)phenol (S1, 1.0 eq) in 60 mL of acetone was added 1-bromooctadecane (1.5 eq) and K_2CO_3 (3.0 eq). After refluxed with vigorously stirring overnight, the reaction mixture was filtrated and washed thoroughly with dichloromethane. The combined organic solutions were then evaporated to give the oil-like crude product, which was purified by column chromatography over silica gel by first using hexane as eluent to wash away the excessive 1-bromooctadecane, and then using hexane/DCM (10 : 1) to give the title compound as a white solid (3.20 g, yield: 65%). FD-MS (8 kV) *m/z*: Calcd for $C_{26}H_{41}F_3N_2O$: 454.3; Found: 454.2 (100%) $[M]^+$. ^1H NMR (300 MHz, CD_2Cl_2, ppm) δ 7.31 (t, *J* = 8.1 Hz, 1H), 6.95 (ddd, *J* = 8.4, 2.5, 0.9 Hz, 1H), 6.77 (d, *J* = 7.7 Hz, 1H), 6.68 (s, 1H), 3.94 (t, *J* = 6.5 Hz, 2H), 1.83 – 1.68 (m, 2H), 1.52 – 1.14 (m, 30H), 0.88 (t, *J* = 6.9 Hz, 3H). ^{13}C NMR (75 MHz, CD_2Cl_2, ppm) δ 160.07, 130.85, 130.61, 124.59, 120.96, 118.95, 116.27, 113.35, 68.83, 32.54, 30.30, 30.26, 30.20, 30.17, 29.97, 29.96, 29.72, 26.54, 23.30, 14.48.

3-(octadecyloxy)benzaldehyde (S9). To a solution of 3-hydroxybenzaldehyde (2.5 g, 1.0 eq, 20 mmol) in 60 mL of acetone was added 1-bromooctadecane (10.0 g, 1.5 eq, 30 mmol) and K_2CO_3 (8.3 g, 3.0 eq, 60 mmol). The mixture was heated to 60 °C with vigorous stirring overnight. After cooling to room temperature, the reaction mixture was filtrated and washed thoroughly with DCM. The combined organic solution was then evaporated to give an oil-like crude product, which was purified by column chromatography over silica gel by first using hexane as eluent to wash away the excessive 1-bromooctadecane, and then using hexane/DCM (4 : 1) to give the title compound as a white solid (6.70 g, yield: 89%). FD-MS (8 kV) *m/z*: Calcd for $C_{25}H_{42}O_2$: 374.3; Found: 374.1 (100%) $[M]^+$. HRMS (ESI) *m/z*: Calcd for $C_{25}H_{42}O_2Na$: 397.3083; Found: 397.3073 $[M + Na]^+$. ^1H NMR (300 MHz, CD_2Cl_2, ppm) δ 9.95 (s, 1H), 7.47 – 7.41 (m, 2H), 7.39 – 7.34 (m, 1H), 7.21 – 7.14 (m, 1H), 4.02 (t, *J* = 6.6 Hz, 2H), 1.91 – 1.69 (m, 2H), 1.52 – 1.14 (m, 30H), 0.88 (t, *J* = 6.9 Hz, 3H). ^{13}C NMR (75 MHz, CD_2Cl_2, ppm) δ 192.59, 138.47, 130.55, 123.39, 122.13, 113.51, 68.95, 32.49, 30.26, 30.22, 30.16, 30.14, 29.93, 29.70, 26.53, 23.26, 14.45.

2,2,2-trifluoro-1-(3-(octadecyloxy)phenyl)ethanol (P-1). The synthesis procedure was following the literature[19]. 3-(Octadecyloxy)benzaldehyde S2 (370 mg, 1.0 eq, 1 mmol) and $TMSCF_3$ (0.200 mL, 1.35 eq, 1.35 mmol) were dissolved in anhydrous DMF (10 mL) in a 50-mL round-bottom flask. To this solution was added K_2CO_3 (20 mg, 15% mol, 0.15 mmol), and the mixture was stirred vigorously at room temperature overnight. The reaction mixture was quenched with 20 mL aqueous solution of HCl (1.0 M), stirred for 1 hour, and then extracted with diethyl ether (3 × 30 mL). The combined

organic layers were finally washed with brine, dried over anhydrous Na_2SO_4, and then concentrated under reduced pressure. The crude product was purified by column chromatography over silica gel with hexane/DCM (3 : 1) as eluent to give the title compound (380 mg, yield: 85%) as a white solid. FD-MS (8 kV) *m/z*: Calcd for $C_{26}H_{43}O_2F_3$ 444.3; Found 444.3 (100%) $[M]^+$. HRMS (ESI) *m/z*: Calcd for $C_{26}H_{43}O_2F_3Na$: 467.3113; Found: 467.3100 $[M + Na]^+$. ^1H NMR (300 MHz, CD_2Cl_2, ppm) δ 7.32 (t, J = 8.2 Hz, 1H), 7.08 – 6.99 (m, 2H), 6.97 – 6.91 (m, 1H), 5.01 (q, J = 6.8 Hz, 1H), 3.97 (t, J = 6.6 Hz, 2H), 2.40 (br, 1H), 1.90 – 1.67 (m, 2H), 1.58 – 1.15 (m, 30H), 0.89 (t, J = 6.6 Hz, 3H). ^{13}C NMR (75 MHz, CD_2Cl_2, ppm) δ 159.96, 136.15, 130.16, 126.83, 123.10, 119.97, 116.03, 114.14, 73.75, 73.32, 72.90, 72.48, 68.75, 32.53, 30.30, 30.27, 30.21, 30.19, 30.00, 29.97, 29.81, 26.59, 23.29, 14.48.

1-(1-chloro-2,2,2-trifluoroethyl)-3-(octadecyloxy)benzene (P-2). To a mixture of P-1 (147 mg, 0.33 mmol) and pyridine (32 mg, 0.40 mmol) was slowly added thionyl chloride (48 mg, 0.40 mmol) and catalytic amount of DMF (5 drops). The mixture was heated at 80 °C for 5 h. After cooling down to room temperature, the mixture was quenched by water and extracted with dichloromethane for three times The combined organic layers were washed with diluted HCl, water, and brine, and then dried over $MgSO_4$. The solvents were removed under reduced pressure and the residue was purified by column chromatography over silica gel (eluent: hexane/DCM = 20 : 1) to give the title compound (96 mg, yield: 63%) as a white solid. FD-MS (8 kV) *m/z*: Calcd for $C_{26}H_{42}ClF_3O$ 462.3; Found: 462.3 (100%) $[M]^+$. ^1H NMR (300 MHz, CD_2Cl_2, ppm) δ 7.32 (t, J = 7.9 Hz, 1H), 7.09 – 7.00 (m, 2H), 6.96 (ddd, J = 8.3, 2.5, 1.0 Hz, 1H), 5.14 (q, J = 6.9 Hz, 1H), 3.97 (t, J = 6.5 Hz, 2H), 1.89 – 1.68 (m, 2H), 1.52 – 1.15 (m, 30H), 0.88 (t, J = 6.9 Hz, 3H). ^{13}C NMR (175 MHz, CD_2Cl_2, ppm) δ 160.02, 133.90, 130.37, 126.47, 124.88, 123.30, 121.71, 121.23, 116.66, 115.48, 68.84, 59.46, 59.27, 59.07, 58.88, 32.53, 30.29, 30.26, 30.20, 30.17, 29.97, 29.77, 26.57, 23.29, 14.47.

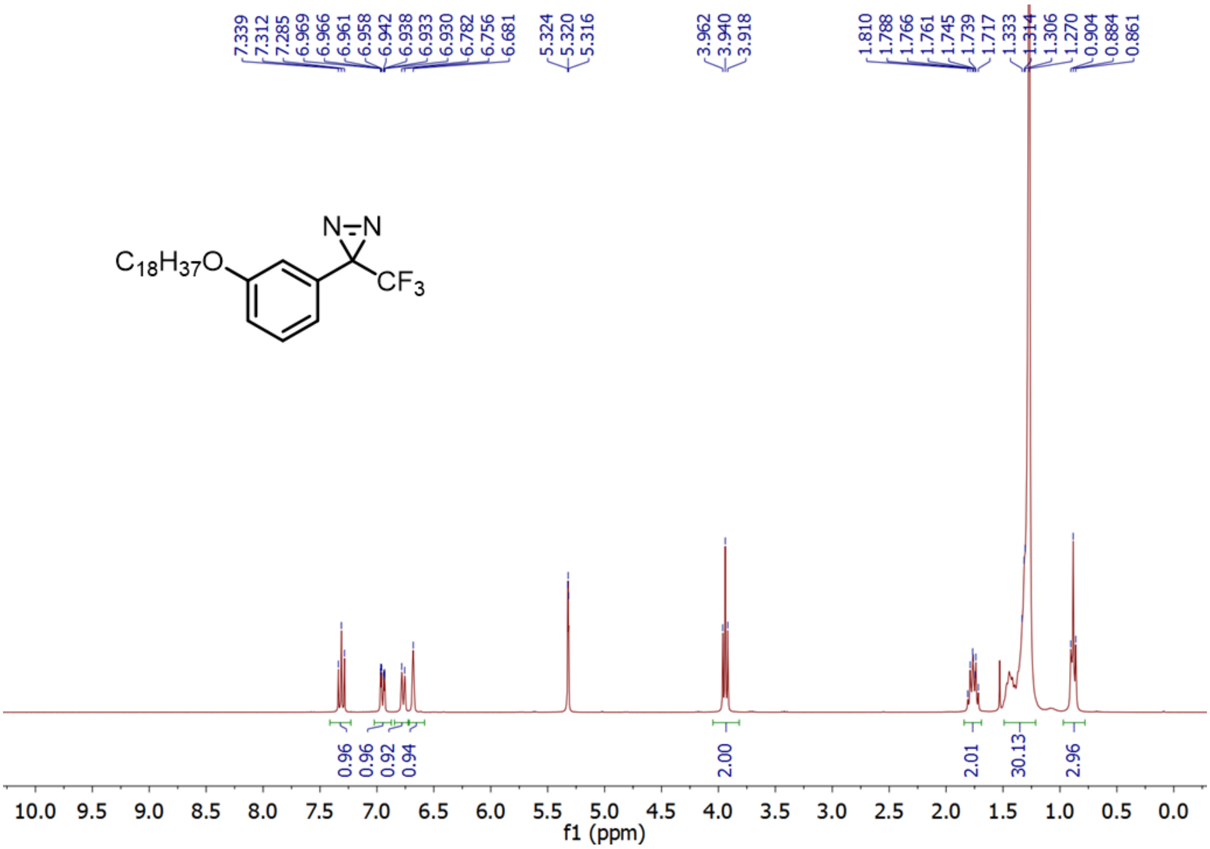

Supplementary Figure 14. ^{1}H nuclear magnetic resonance characterization of MBB-1. ^{1}H nuclear magnetic resonance spectroscopy analysis of MBB-1 (300 MHz, CD_2Cl_2). Inset: chemical structure of MBB-1.

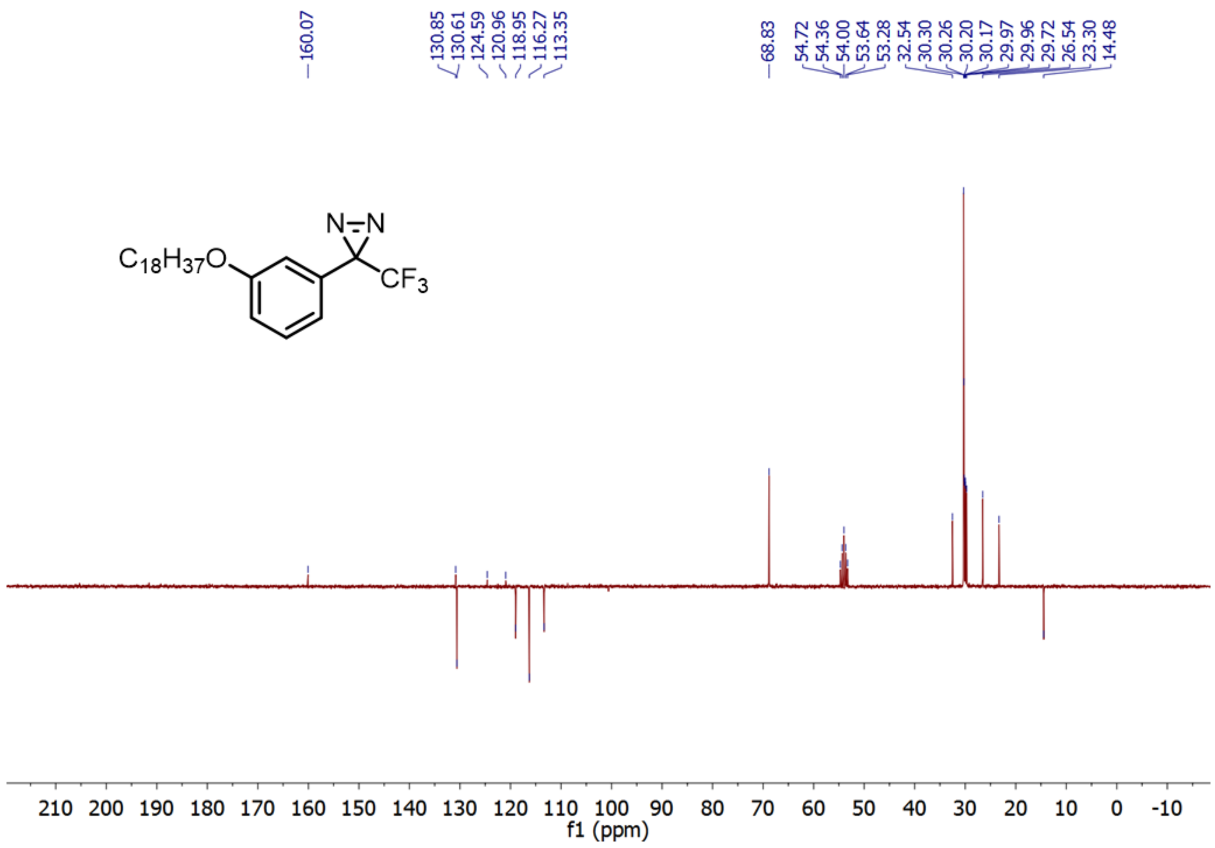

Supplementary Figure 15. ^{13}C nuclear magnetic resonance characterization of MBB-1. ^{13}C nuclear magnetic resonance spectroscopy analysis of MBB-1 (75 MHz, CD_2Cl_2). Inset: chemical structure of MBB-1.

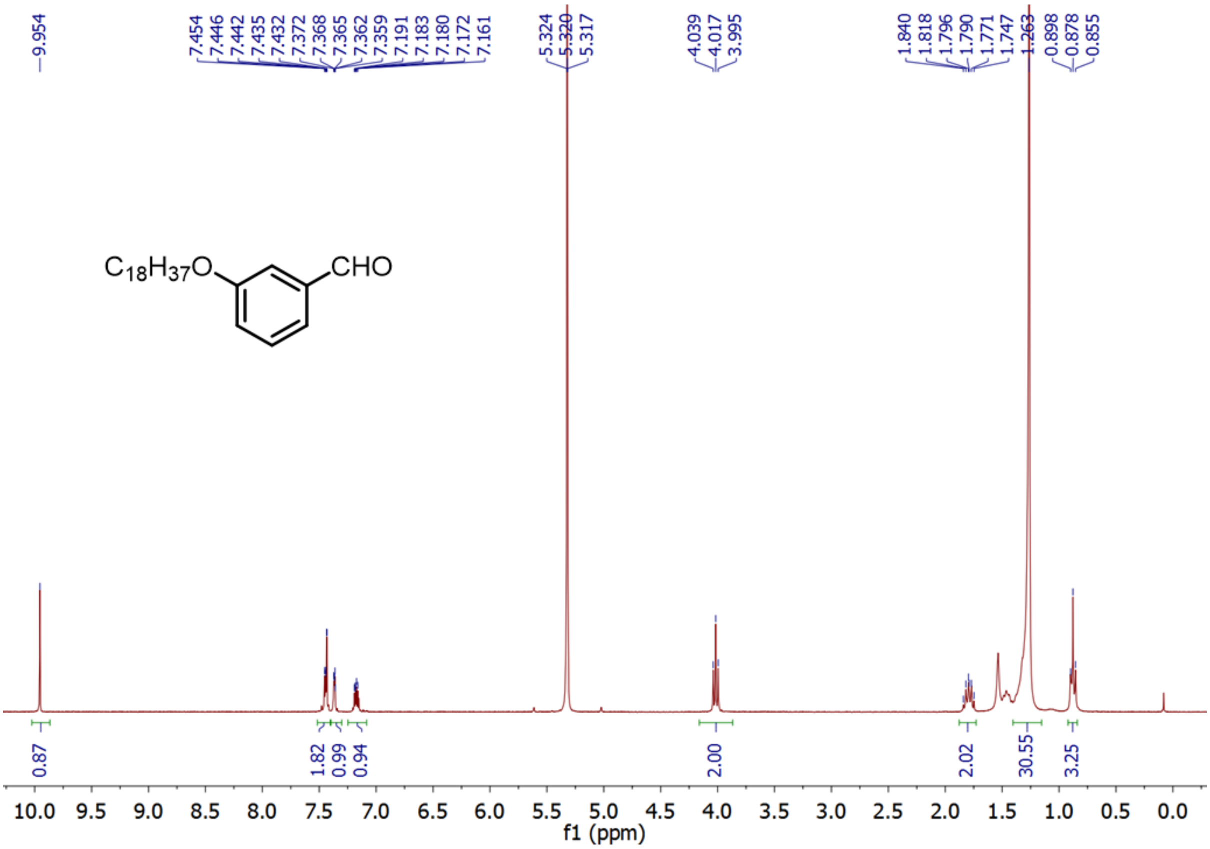

Supplementary Figure 16. ^{1}H nuclear magnetic resonance characterization of S9. ^{1}H nuclear magnetic resonance spectroscopy analysis of S9 (300 MHz, CD_2Cl_2). Inset: chemical structure of S9.

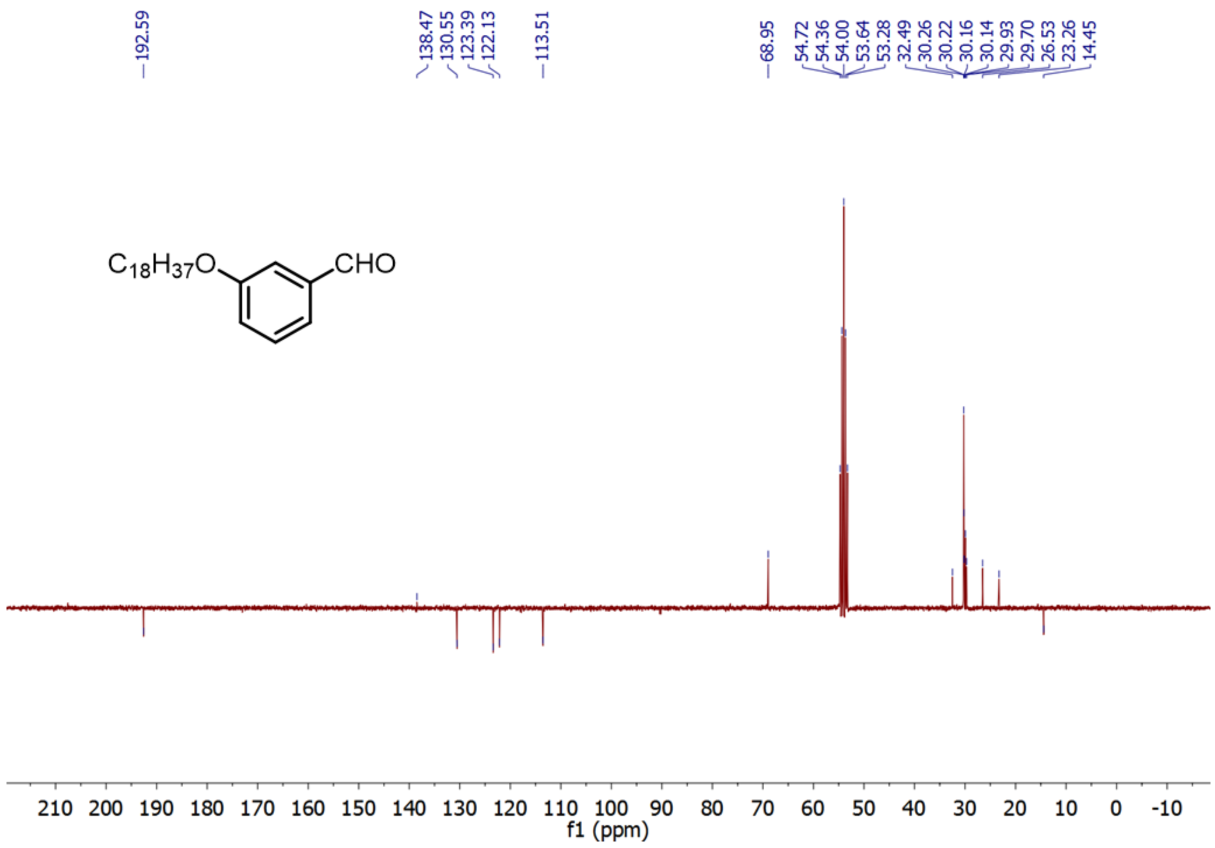

Supplementary Figure 17. ^{13}C nuclear magnetic resonance characterization of S9. ^{13}C nuclear magnetic resonance spectroscopy analysis of S9 (75 MHz, CD_2Cl_2). Inset: chemical structure of S9.

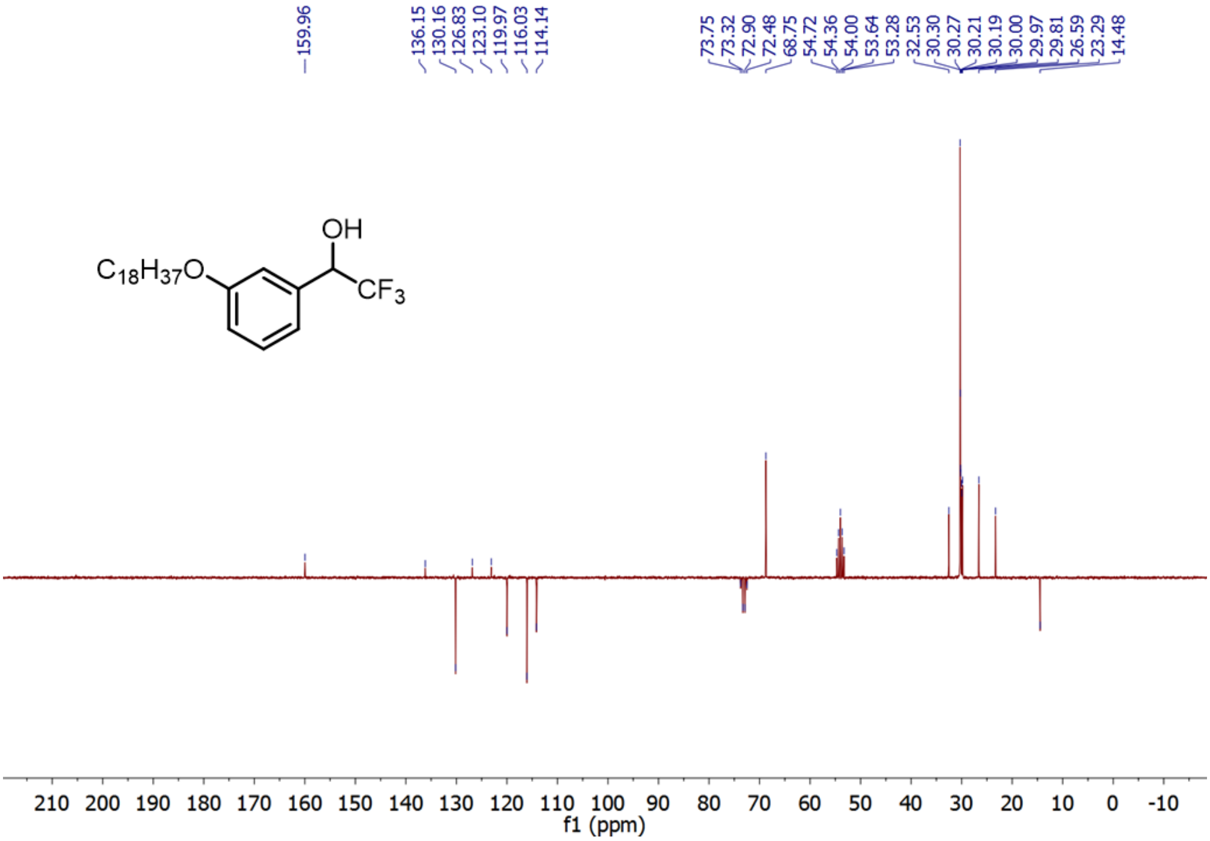

Supplementary Figure 18. ^{13}C nuclear magnetic resonance characterization of P-1. ^{13}C nuclear magnetic resonance spectroscopy analysis of P-1 (75 MHz, CD_2Cl_2). Inset: chemical structure of P-1.

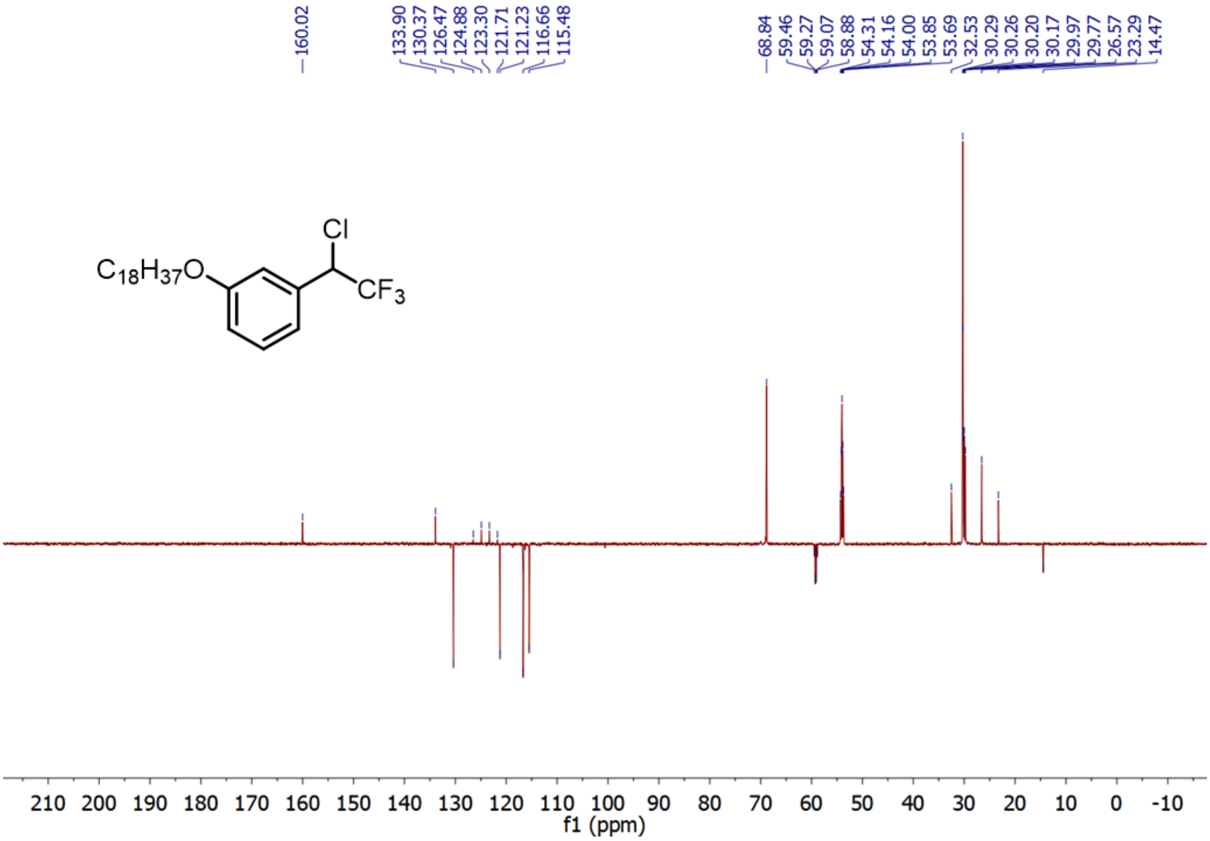

Supplementary Figure 19. ^{13}C nuclear magnetic resonance characterization of P-2. ^{13}C nuclear magnetic resonance spectroscopy analysis of P-2 (175 MHz, CD_2Cl_2). Inset: chemical structure of P-2.

Supplementary references

1. Balderschi, A., Baroni, S. & Resta, R. Band offsets in lattice-matched heterojunctions: A model and first-principles calculations for GaAs/AlAs. *Phys. Rev. Lett.* **61,** 734–737 (1988).
2. Coletti, C. *et al.* Charge neutrality and band-gap tuning of epitaxial graphene on SiC by molecular doping. *Phys. Rev. B* **81,** 235401 (2010).
3. Yan, J., Zhang, Y., Kim, P. & Pinczuk, A. Electric field effect tuning of electron-phonon coupling in graphene. *Phys. Rev. Lett.* **98,** 166802 (2007).
4. Froehlicher, G. & Berciaud, S. Raman spectroscopy of electrochemically gated graphene transistors: Geometrical capacitance, electron-phonon, electron-electron, and electron-defect scattering. *Phys. Rev. B - Condens. Matter Mater. Phys.* **91,** 205413 (2015).
5. Samuels, A. J. & Carey, J. D. Molecular doping and band-gap opening of bilayer graphene. *ACS Nano* **7,** 2790–2799 (2013).
6. Wehling, T. O. *et al.* Molecular doping of graphene. *Nano Lett.* **8,** 173–177 (2008).
7. Yu, Y.-J. *et al.* Tuning the graphene work function by electric field effect. Supplementary Information. *Nano Lett.* **9,** 3430–3434 (2009).
8. Ha, T.-J. *et al.* Transformation of the Electrical Characteristics of Graphene Field- Effect Transistors with Fluoropolymer. *ACS Appl. Mater. Interfaces* **5,** 16–20 (2013).
9. Besler B. H., Merz K. M., J. & A., K. P. Atomic charges derived from semiempirical methods. *J. Comput. Chem.* **11,** 431–439 (1990).
10. Frisch, M. J. *et al.* Gaussian 09, Revision D.01. Gaussian, Inc., Wallingford, CT, 2009.
11. Soler, J. M. *et al.* The SIESTA method for ab initio order-N materials simulation. *J. Phys. Condens. Matter* **14,** 2745-2779 (2002).
12. Cornil, D., Van Regemorter, T., Beljonne, D. & Cornil, J. Work function shifts of a zinc oxide surface upon deposition of self-assembled monolayers: a theoretical insight. *Phys. Chem. Chem. Phys.* **16,** 20887–20899 (2014).
13. Christodoulou, C. *et al.* Tuning the work function of graphene-on-quartz with a high weight molecular acceptor. *J. Phys. Chem. C* **118,** 4784–4790 (2014).
14. Heimel, G., Romaner, L., Br??das, J. L. & Zojer, E. Organic/metal interfaces in self-assembled monolayers of conjugated thiols: A first-principles benchmark study. *Surf. Sci.* **600,** 4548–4562 (2006).
15. D'Avino, G., Muccioli, L., Zannoni, C., Beljonne, D. & Soos, Z. G. Electronic polarization in organic crystals: A comparative study of induced dipoles and intramolecular charge redistribution schemes. *J. Chem. Theory Comput.* **10,** 4959–4971 (2014).
16. D'Avino, G., Vanzo, D. & Soos, Z. G. Dielectric properties of crystalline organic molecular films in the limit of zero overlap. *J. Chem. Phys.* **144,** 34702 (2016).

17. Kim, K. & Jordan, K. D. Comparison of Density Functional and MP2 Calculations on the Water Monomer and Dimer. *J. Phys. Chem.* **98,** 10089–10094 (1994).

18. Lawrence, E. J. *et al.* 3-Aryl-3-(trifluoromethyl)diazirines as Versatile Photoactivated 'Linker' Molecules for the Improved Covalent Modification of Graphitic and Carbon Nanotube Surfaces. *Chem. Mater.* **23,** 3740–3751 (2011).

19. Prakash, G. K. S. *et al.* Facile synthesis of TMS-protected trifluoromethylated alcohols using trifluoromethyltrimethylsilane (TMSCF3) and various nucleophilic catalysts in DMF. *J. Org. Chem.* **71,** 6806–6813 (2006).
